# QTL mapping for growth-related traits by constructing the first genetic linkage map in Simao pine

**DOI:** 10.1186/s12870-022-03425-y

**Published:** 2022-01-22

**Authors:** Dawei Wang, Lin Yang, Chen Shi, Siguang Li, Hongyan Tang, Chengzhong He, Nianhui Cai, Anan Duan, Hede Gong

**Affiliations:** 1 Key Laboratory for Forest Resource Conservation and Utilization in the Southwest Mountains of China, Ministry of Education, Southwest Forestry University, Kunming, China; 2 Key Laboratory for Forest Genetic and Tree Improvement & Propagation in Universities of Yunnan Province, Southwest Forestry University, Kunming, China; 3 Yunnan Academy of Forestry, Kunming, China; 4 Puer City Institute of Forestry Sciences, Puer, China; 5 School of Geography, Southwest Forestry University, Kunming, China

**Keywords:** QTL mapping, growth-related traits, genetic linkage map, Simao pine, SLAF-seq

## Abstract

**Background:**

Simao pine is one of the primary economic tree species for resin and timber production in southwest China. The exploitation and utilization of Simao pine are constrained by the relatively lacking of genetic information. Construction a fine genetic linkage map and detecting quantitative trait locis (QTLs) for growth-related traits is a prerequisite section of Simao Pine's molecular breeding program.

**Results:**

In our study, a high-resolution Simao pine genetic map employed specific locus amplified fragment sequencing (SLAF-seq) technology and based on an F_1_ pseudo-testcross population has been constructed. There were 11,544 SNPs assigned to 12 linkage groups (LGs), and the total length of the map was 2,062.85 cM with a mean distance of 0.37 cM between markers. According to the phenotypic variation analysis for three consecutive years, a total of seventeen QTLs for four traits were detected. Among 17 QTLs, there were six for plant height (Dh.16.1, Dh16.2, Dh17.1, Dh18.1–3), five for basal diameter (Dbd.17.1–5), four for needle length (Dnl17.1–3, Dnl18.1) and two for needle diameter (Dnd17.1 and Dnd18.1) respectively. These QTLs individually explained phenotypic variance from 11.0–16.3%, and the logarithm of odds (LOD) value ranged from 2.52 to 3.87.

**Conclusions:**

In our study, a fine genetic map of Simao pine applied the technology of SLAF-seq has been constructed for the first time. Based on the map, a total of 17 QTLs for four growth-related traits were identified. It provides helpful information for genomic studies and marker-assisted selection (MAS) in Simao pine.

**Supplementary Information:**

The online version contains supplementary material available at 10.1186/s12870-022-03425-y.

## Background

*Pinus kesiya *Royle ex Gordon var. *langbianensis* (A. Chev.) Gaussen (2*n* = 24) [64], also called Simao/Szemao pine in China, is a geographic variant of *Pinus kesiya* [54, 55], mainly distributed in the humid and sub-humid mountainous areas in southwest China [30, 74]. Compared with other conifer tree species, it has unique rapid growth characteristics that its branches can grow 2 or more rounds a year [41, 66]. It is one of the most important timber and resin production tree species in Yunnan Province [5]. Simao pine is not only a high commercial value plant for its higher turpentine output [13, 57] but also essential tree species in the forest ecosystem for the function of biological carbon sequestration and water conservation [6, 29, 58, 73]. However, its exploitation and utilization are constrained by relatively lacking genetic information [5].

Highly saturated genetic linkage map construction is a useful tool for QTL mapping and MAS [19, 71, 72]. During the last two decades, a large number of genetic maps for perennial woody plants had constructed [18, 26, 52], various QTLs associated with essential traits had been identified based on these maps [12], [42] [36]. However, a great majority of them were low saturated frame-work maps that lowered the degree of accuracy of QTL mapping [28, 31]. Single nucleotide polymorphism (SNP) is a sort of molecular marker technique developed by high-throughput sequencing. It is convenient, abundant, highly polymorphic and commonly used in genetic map construction [34, 45, 46]. In pace with the rapid development technology of next-generation sequencing (NGS), the technology of SLAF-seq becomes one of the popular methods for SNP markers development and high-resolution genetic map construction [9, 44, 69]. Until now, various plants genetic linkage maps had established by SLAF-seq, and it greatly heighten the efficiency and the degree of QTL mapping accuracy [11, 37, 68, 70].

Construction of genetic linkage map for Simao pine will provide helpful information for genomic studies and facilitate the breeding applications. Growth-related traits were important economic traits for woody tree breeding, and detecting QTLs for these traits is a crucial section in the molecular breeding program for Simao Pine. Therefore, we employed the SLAF-seq technology to actualize the fast SNPs development and a high-density linkage map will be constructed. The QTLs linked to growth-related traits will be identified based on the genetic linkage map. It will provide a powerful tool for future detection of other economic characteristics QTLs and MAS in Simao pine breeding.

## Results

### Analysis of sequencing data and SLAF markers

**Fig. 1:**
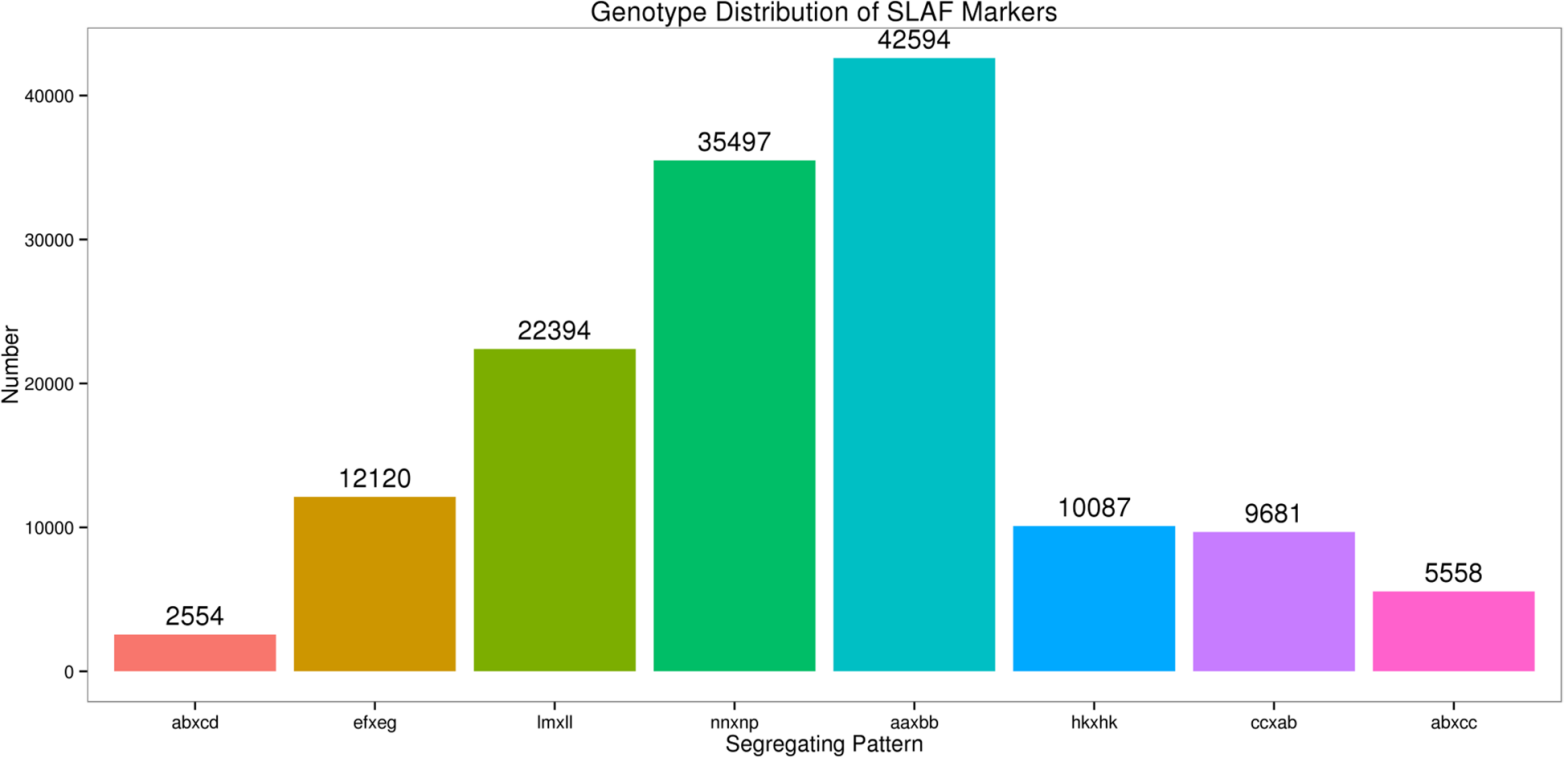
Segregation pattern of polymorphic SLAF markers. The x-axis represents the segregating pattern numbers and the y-axis indicates the genotypes of markers

The Simao pine SLAF libraries were constructed successfully. A total of 461.41 M reads (guanine-cytosine of 40.23% and Q30 of 92.25%) were obtained. The number of reads for the maternal and paternal parents was 22,947,158 and 18,534,664, the mean for the F_1_ individual was 4,659,126 (Table 1). After filtering out the low-quality reads, the number of SLAFs for the two parents and average in F1 progeny was 535,598, 482,851, and 375,315. The average depth of the SLAFs for the maternal and paternal parent was 10.48-fold and 9.77-fold, and the average for each F1 individual was 3.47-fold (Table 1). A total of 633,086 high-quality SLAFs were obtained. Among these markers, 239,790 were polymorphic markers (37.88%), 385,976 were non-polymorphic markers (60.97%), and 7,320 were repetitive markers (1.15%). Finally, 140,485 polymorphic SLAFs of 8 segregation patterns were achieved (Fig. 1). After removing the markers showing aa × bb segregation pattern, 97,891 (15.46%) polymorphic SLAFs will be used to further Simao pine genetic map construction.

**Table 1:** Statistics for sequenced data

Sample ID	Total Reads	Q30 percentage (%)	GC percentage (%)	SLAF Number	Average Depth
SM11	22,947,158	91.72	40.27	535,598	10.48
JG1	18,534,664	91.57	39.91	482,851	9.77
offspring	4,659,126	92.25	40.23	375,315	3.47

### Construction and evaluation of genetic linkage map

**Fig. 2:**
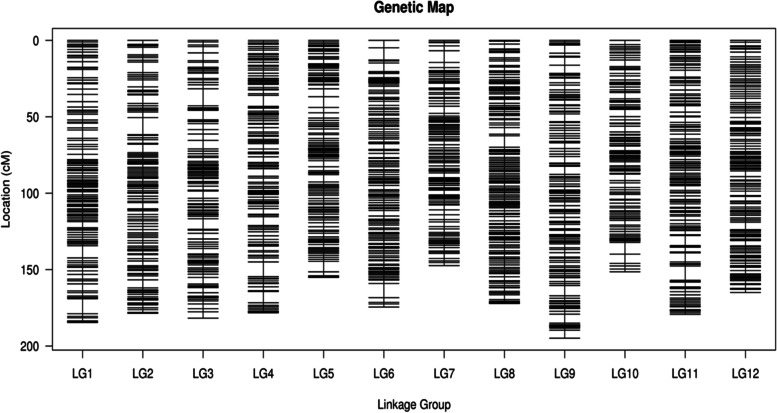
The high-density linkage map of Simao pine. A black bar indicates a SLAF marker. The x-axis represents the linkage group number and the y-axis indicates the genetic distance (cM) within each linkage group

After discarding the unsuitable markers, a total of 5,643 SLAFs were used successfully for the linkage map construction. Among them, 11,544 SNP markers were detected (Table 2). Based on these markers, we constructed a high saturated genetic linkage map covering 2062.85 cM, comprising 12 LGs, and a mean distance of 0.37 cM (Fig. 2, Table 2). The genetic length of individual LGs varied from 147.38 (LG4) to 194.85 cM (LG9) with a mean of 171.90 cM. Among 12 LGs, LG8 was the largest linkage group (523 SLAFs), while LG7 was the smallest group (367 SLAFs). The average number of markers for each LG was 470. For the density, LG5 was the densest linkage group with the minimum marker distance (0.32 cM), whereas LG1, LG3, and LG7 were the lowest density linkage groups (0.40 cM). The max gap in the map was 11.17 cM located in LG2 and LG3.

**Table 2:** Marker information for high-density genetic map

LGs	SLAFs Number	Total Distance (cM)	Average Distance (cM)	Max Gap (cM)	SNPs Number	Trv	Tri	Trv/Tri
LG1	458	184.59	0.40	9.76	918	291	627	0.46
LG2	467	178.60	0.38	11.17	962	302	660	0.46
LG3	452	181.78	0.40	11.17	938	275	663	0.41
LG4	513	178.28	0.35	9.64	1,019	334	685	0.49
LG5	492	155.06	0.32	7.03	1,003	295	708	0.42
LG6	468	174.50	0.37	9.26	934	311	623	0.50
LG7	367	147.38	0.40	7.66	698	207	491	0.42
LG8	523	172.15	0.33	7.53	1,062	333	729	0.46
LG9	508	194.85	0.38	6.71	988	324	664	0.49
LG10	384	151.42	0.40	7.71	882	302	580	0.52
LG11	516	179.28	0.35	9.31	1,063	352	711	0.50
LG12	495	164.96	0.33	4.89	1,077	318	759	0.42
Total	5,643	2,062.85	0.37	11.17	11,544	3,644	7,900	0.46

Trv and Tri indicate the transversion and transition numbers, respectively

**Fig. 3:**
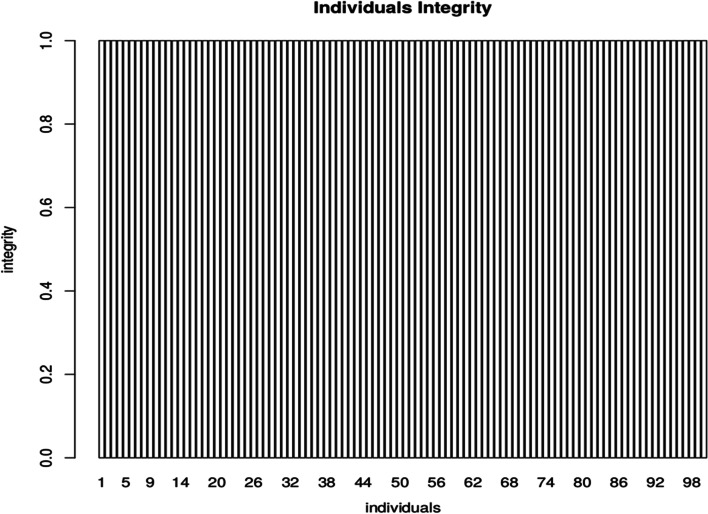
The integrity distribution map of all individuals. The x-axis represents the 100 individuals and y-axis represents the complete degree of mapped markers

Three approaches were used for detecting the quality of Simao pine genetic map. (1) The markers integrity analysis showed that each individual mapped marker's complete degree was 99.99% (Fig. 3), the average depth for parents was more than five times of the offspring (Table 3). It suggested that genotyping was accurate and the mapping population was suitable for further analysis. (2) The result of Haplotype maps analysis revealed that most of the recombination blocks were distinctly defined (Supplementary Fig. 1). It suggested that the constructed high saturated genetic map was adaptive for subsequent genetic analysis. (3) The analysis of Heat maps showed that the markers were well ordered in most linkage groups, indicated that the constructed Simao pine genetic map with high accuracy (Supplementary Fig. 2).

**Table 3:** Statistics of the mapped marker depth

Sample ID	Marker Number	Total Depth	Average Depth
SM11	5,643	243,344	43.12
JG1	5,643	220,795	39.13
Offspring	5,425	43,439	8.01

### Phenotypic variation analysis

**Fig. 4:**
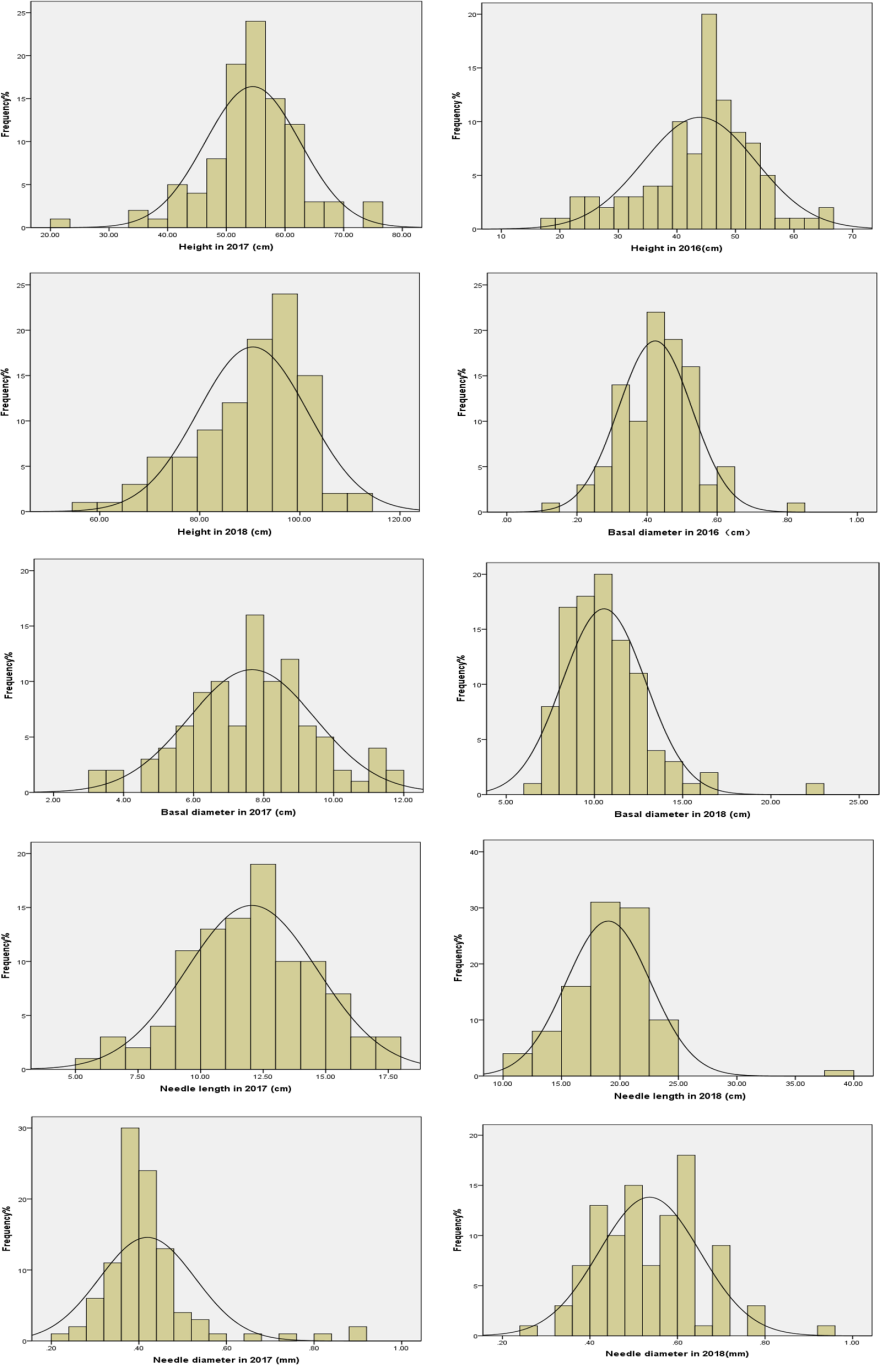
Frequency distributions of growth traits in mapping population during three consecutive years

The 3 years phenotypic data and statistical values for growth-related traits were summarized (Table 4). The results showed that the four traits were normal distribution for three years (Fig. 4). And a relatively higher degree of genetic variation was found. The CV of plant height was 21.74%, 14.80% and 12.07% during 2016, 2017 and 2018. The CV of basal diameter was 25.21% (2016), 23.42% (2017), and 22.33% (2018) respectively. The CV of needle length was 21.69% (2017) and 18.89% (2018). The CV of needle diameter was 25.96% (2017) and 21.44% (2018). The Pearson correlations analyses showed the significant correlation among four traits (Table 5).

**Table 4:** Statistics of growth traits of mapping population during three consecutive years

Traits	Average	Min	Max	SD	CV (%)
Height 2016 (cm)	43.89	18.4	68.7	9.54	21.74
Height 2017 (cm)	54.49	22.3	76.5	8.07	14.8
Height 2018 (cm)	90.59	57.2	113.5	10.93	12.07
Basal diameter 2016 (mm)	0.42	0.12	0.8	0.11	25.21
Basal diameter 2017 (mm)	7.66	3.26	11.65	1.79	23.42
Basal diameter 2018 (mm)	10.54	6.78	22.43	2.35	22.33
Needle length 2017 (cm)	12.05	5.23	17.91	2.61	21.69
Needle length 2018 (cm)	19	11.61	39.39	3.59	18.89
Needle diameter 2017 (mm)	0.42	0.24	0.92	0.11	25.96
Needle diameter 2018 (mm)	0.54	0.27	0.93	0.11	21.44

**Table 5:** The correlation coefficients between 4 traits

	PH2016	BD2016	PH2017	BD2017	NL2017	ND2017	PH2018	BD2018	NL2018	ND2018
PH2016	1									
BD2016	0.695^b^	1								
PH2017	0.755^b^	0.578^b^	1							
BD2017	0.246^a^	0.178	0.360^b^	1						
NL2017	0.269^b^	0.186	0.366^b^	0.611^b^	1					
ND2017	0.302^b^	0.323^b^	0.375^b^	0.287^b^	0.320^b^	1				
PH2018	0.595^b^	0.389^b^	0.703^b^	0.582^b^	0.514^b^	0.290^b^	1			
BD2018	0.372^b^	0.223^a^	0.454^b^	0.663^b^	0.447^b^	0.307^b^	0.595^b^	1		
NL2018	0.439^b^	0.358^b^	0.480^b^	0.453^b^	0.412^b^	0.436^b^	0.530^b^	0.603^b^	1	
ND2018	0.308^b^	0.314^b^	0.345^b^	0.288^b^	0.275^b^	0.373^b^	0.414^b^	0.277^b^	0.481^b^	1

^a^significant at *p* < 0.05, ^b^significant at *p* < 0.01

### QTL mapping

Using the constructed map and analyzing the data of phenotypic characteristics in the mapping population, 17 QTLs linked to 4 traits were identified (Table 6, Supplementary Fig. 3). The individual QTL explained the phenotypic variation varied from 11.0–16.3%, and the LOD value ranged from 2.52 to 3.87. There were 6 plant height QTLs detected in the map. In 2016, two plant height QTLs were located on LG3 (Dh16.1) and LG10 (Dh16.2) explained 12.7% and 12.4% of the phenotypic variance. In 2017, only one plant height QTL located on LG9 (Dh17.1) explained 15.5% of the phenotypic variation was detected. In 2018, the other three plant height QTLs were identified which located on LG11 (Dh18.1, Dh18.2), and LG12 (Dh18.3), and explained 16.3%, 16.1% and 13.8% of the phenotypic variation, respectively. A total of 5 QTLs associated with basal diameter were detected on LG3 (Ddb17.1, 11.6%), LG4 (Ddb17.2, 12.7%) and LG6 (Ddb17.3, 11.6%; Ddb17.4, 13.1%; Ddb17.5, 11.4%), respectively. Four needle length QTLs were identified which located on LG1 (Dnl17.1, 12.1%; Dnl17.2, 11.0%; Dnl17.3, 12.1%) and LG12 (Dnl18.1, 13.5%), respectively. There were two needle diameter QTLs located on LG4 (Dnb17.1 and Dnb18.1) explained 12.9% and 13.1% of the phenotypic variation were detected.

**Table 6:** Quantitative trait loci (QTLs) for growth-related traits during 3 years in the mapping population

Trait	Year	LG	QTLs	Marker	Peak Position (cM)	Confidence Interval (cM)	LOD	Explained Variance (%)
Plant Height	2016	LG3	Dh16.1	Marker162009	29.068	18.774–45.722	2.94	12.7
		LG10	Dh16.2	Marker186661	49.885	49.885–54.091	2.87	12.4
	2017	LG9	Dh17.1	Marker111742	58.881	53.171–60.966	3.66	15.5
	2018	LG11	Dh18.1	Marker90236	27.819	24.054–38.14	3.87	16.3
		LG11	Dh18.2	Marker120973	56.742	56.237–58.268	3.82	16.1
		LG12	Dh18.3	Marker31588	18.009	16.999–20.409	3.23	13.8
Basal Diameter	2017	LG3	Dbd17.1	Marker110266	170.435	169.425–170.435	2.67	11.6
		LG4	Dbd17.2	Marker69728	137.585	130.262–138.461	2.95	12.7
		LG6	Dbd17.3	Marker171746	82.624	82.624–84.708	2.66	11.6
		LG6	Dbd17.4	Marker92854	89.329	85.214–89.329	3.04	13.1
		LG6	Dbd17.5	Marker145277	104.629	102.175–105.122	2.62	11.4
Needle Length	2017	LG1	Dnl17.1	Marker36587	7.716	7.682–7.822	2.79	12.1
		LG1	Dnl.17.2	Marker170751	27.84	26.017–27.84	2.52	11.0
		LG1	Dnl17.3	Marker129013	95.986	95.481–98.522	2.80	12.1
	2018	LG12	Dnl18.1	Marker52737	16.999	16.689–17.121	3.14	13.5
Needle Diameter	2017	LG4	Dnd17.1	Marker30236	154.595	154.137–154.616	3.00	12.9
	2018	LG4	Dnd18.1	Marker160818	171.684	171.351–171.752	3.05	13.1

## Discussion

Construction of the high-resolution map for Simao pine will provide helpful information for genomic studies and facilitate the breeding applications. In our study, a fine genetic map of Simao pine applied the technology of SLAF-seq has been constructed. It contained 12 LGs and 11,544 SNPs spanned 2,062.85 cM with a mean marker distance of 0.37 cM, representing a significant improvement over the previous linkage maps in coniferous plants [8, 10, 14, 15, 39, 60]. As we know, this was one of the highest saturated genetic maps to date in coniferous tree species. Furthermore, a total of 17 QTLs for four growth-related traits were identified based on the constructed genetic map, and these QTLs were valuable resources for genetic breeding and MAS in Simao pine.

An appropriate mapping population laid a solid foundation for the genetic map construction [75]. It's hard for perennial woody trees to get Backcross (BC), Recombination Inbred Lines (RILs) and F_2_ populations in the short term because of the long generation constraints. The pseudo-testcross strategy has been put forward that the F_1_ population was created to replace the other populations [20]. This strategy has been successfully applied to various forestry trees, especially non-model and un-sequenced species [32, 35, 47, 61, 62, 65]. In this report, nine F_1_ populations were obtained by artificial hybridization, based on the analysis of field phenotypic characteristics variation among populations and genetic similarity coefficient among parents, superior clones SM11 (high resin content) and JG1 (fast growth) were chosen for maternal and paternal parents. The F_1_ hybrid population was applied as the mapping population for map construction in our study. The obvious variation will present in the segregation population due to the significant difference in the resin content and the parents' growth speed, which could facilitate QTL mapping for these traits.

Molecular markers were powerful tools for genetic map construction [2]. The mainstream molecular markers for genetic linkage map construction of heterozygous perennial forest tree species included SNP, simple sequence repeat (SSR), inter-simple sequence repeat (ISSR), amplified fragment length polymorphism (AFLP) and random amplified polymorphic DNA (RAPD) et al. [7, 21, 28, 31, 38, 56]. Among these markers, SNP was thought of as one of the ideal markers for genetic map construction for the merits of abundance, fast and covering the whole genome [3, 16]. Significant changes have taken place in genetic map construction with the development of high-throughput sequencing technology [15]. Recently, SLAF-seq technique has become one of the most popular SNP marker development assays [45]. A high-density genetic linkage map for Simao pine had been successfully constructed by using this approach. It indicated that SNP markers could be efficiently applied in constructing a genetic linkage map of Simao pine.

High-quality genetic maps can increase the accuracy of QTL mapping [28, 31, 32, 35]. The number of markers in the genetic map is one of the essential indicators to evaluate its quality. A genetic map with a large number of markers has the characteristics of suitable distance and high-resolution [70]. This constructed genetic map was the first map that contained over ten thousand SNP markers in coniferous tree species. It supported that we have built a high-quality genetic map for Simao Pine. Moreover, other three approaches were used for evaluating the quality of Simao pine genetic map. All results indicated that the current high-accuracy map would provide sufficient information for QTL mapping.

Growth-related traits were important economic traits for woody tree breeding, and detecting QTLs for growth-related traits is an introductory section in the molecular breeding program for Simao Pine. In this study, a total of 17 QTLs for four growth-related traits were identified. The individual QTL explained the phenotypic variation varied from 11.0% to16.3%, and it indicated that several significant useful genes might control the growth-related traits of Simao pine (Rönnberg et al., 2005; [28, 31]. In the four traits, only the QTLs for plant height were consistently detected during the three years, but QTLs for the other three traits were not consecutively expressed. It suggested that different genes/QTLs might influence Simao pine's growth-related traits in different seasons/ages or that the QTLs stabilization varied by the effect of environmental change. In agreement with previous studies in the woody tree, growth-related traits were mainly quantitative traits, which dominated by involved genes and easily affected by the environment, probably changes as the tree matures [27, 67]. In other words, the multi-environment QTL analysis is more accurate than a single-environment experiment for the heterozygous perennial woody tree growth-related traits QTL mapping [17]. Thus, to eliminate interference brought by the environment and improve QTLs accuracy, the multi-environment QTL test in other domains using different backgrounds for Simao pine must be carried out in the future [1, 28, 31].

The size of the mapping population decided the success of genetic mapping and QTL analysis, and the influence of missing genotypes is more obvious in small-sized populations than in large one. In general, mapping population consisting of 50–250 individuals may be sufficient to construct the initial skeletal linkage map [24], [4]. However, a larger population size is needed for high resolution or fine mapping [40, 54, 55]. In our study, a main limitation of the Simao pine genetic linkage map is that it is smaller mapping population size (100 individuals), it may be provided fragmented linkage groups and inaccurate locus order for the genetic linkage map and affected the accuracy of the QTL mapping [40]. It is well known that artificially controlled pollination of conifers trees is more difficult and the number of hybrid offspring is less than other tree species too [23]. To our knowledge, high-density genetic maps which constructed with the small size mapping populations have been successfully used for QTL fine mapping [61, 62, 75]. However, for further improving the accuracy of the map and QTLs, the size of Simao pine mapping population must be increased in the future.

## Conclusions

We report the first high-density genetic map for Simao pine. The map was constructed using an F_1_ population and was based on SNP markers developed by using the SLAF-seq approach, which allowed the efficient development of a large number of markers in a short time. A total of 17 QTLs for growth-related traits were identified based on the constructed genetic map. The results of this study will provide a platform for map-based gene isolation and molecular breeding for Simao pine.

## Methods

### Mapping population and DNA extraction

According to factorial mating design, eleven superior clones with the good characters of rapid growth and high resin content were selected as the hybrid parents from 105 clones. Among them, five clones as the male parents (superior clone JG1, NR7, LC3, ZY1, PW2) and the other six clones for the female parents (PW12, LC9, JG7, JD5, PW3, SM11). In the spring of 2014, a total of 30 hybridized combinations of artificially controlled pollination were conducted. Two years later, a total of 9 full-sib families were obtained and grown at the farm of Pu'er city institute of forestry sciences (N 22◦ 47′/E 100◦ 59′) by harvesting, sowing and culturing the seedlings. Proceed to the next step, family 9 was selected as the mapping population for Simao pine genetic linkage map construction by analyzing population phenotypic variation and parent's genetic similarity coefficient [57]. The parents for the mapping population were superior clone SM11 (maternal, with the characteristic of high resin content) and JG1 (paternal, with the aspect of rapid growth). Fresh and young healthy needles from parents and mapping population (100 hybrid individuals) were collected and frozen in liquid nitrogen at once. The genomic DNA was isolated using the improved cetyl trimethyl ammonium bromide (CTAB) method [53]. The permission of the plant materials collection was approved by Southwest Forestry University (project number: 2018Y31). The plant species was identified by *Assoc. Prof.* Jianghua Liu (College of Forestry, Southwest Forestry University), and the voucher specimens were stored at the Key Laboratory for Forest Resources Conservation and Utilization in the Southwest Mountains of China Ministry of Education, Southwest Forestry University, Kunming, China.

### SLAF library establishment and sequencing

The similar experiment procedure of high-throughput sequencing and establishment of the SLAF library for the mapping population was performed according to the previous study by Zhang et al. [68] with minor modified. Briefly, two different steps were applied. First, all of the genomic DNA for SLAF library construction were digested by a single enzyme *Hae* III (New England Biolabs, NEB, USA). Second, only the SLAF fragments in which the length ranging from 414 to 464 bp will be excised and diluted for pair-end sequenced by Illumina HiSeq 2500 platform (Illumina, Inc; San Diego, CA, USA).

### Analysing and genotyping for sequence data

The SLAF-seq data grouping and SNP genotyping were the same as Wang et al. [56]. After discarding the low-quality reads, the remaining reads with more than 90% similarity will gather in the same SLAF locus. As Simao pine is a diploid plant, so only the SLAF has 2 to 4 alleles that will designate as the potential and polymorphic marker. The aa × bb segregation pattern markers will not be used to construct the genetic map, as the mapping population is obtaining by a cross between two heterozygote parents of Simao pine [22, 25].

### Linkage map construction and evaluation

The HighMap software [22] with the cross-pollination (CP) option was utilized for Simao pine genetic linkage map construction. The estimation parameters were set for a minimum LOD threshold of 5.0 for linkage groups and a maximum recombination fraction of 0.4, and the map distance in centi-Morgans was calculated with the Kosambi mapping algorithm [51]. After linkage grouping, the maximum likelihood method was used to order the SLAFs markers in all LGs [50], [37]. The SMOOTH algorithm was utilized to put correct genotyping errors [49]. To evaluate the quality of the constructed Simao pine genetic map, the analysis of mapped markers integrity, construction of haplotype maps and heat maps for each LG were carried out [33, 59, 63].

### Growth-related traits assessment and QTL analysis

Growth-related traits, including plant height, basal diameter, needle length, and needle diameter of the progenies are determined during three consecutive years. The plant height and needle length were measured by a line tape, while the basal diameter and needle diameter were measured with the vernier caliper in December from 2016–2018. The phenotypic variation analysis, including coefficients of variation (CV) and the correlation coefficients between all investigated traits, was performed with software SPSS 20.0. The QTLs underlying the growth-related traits were implemented in MapQTL 6.0 software and the interval mapping method [48]. The 95% Bayesian credible interval method was used to calculate the confidence intervals for all QTLs [43]. One thousand permutations decided the threshold value. According to the permutations, the minimum LOD score of 2.5 was conducted in our study. The percentage of phenotypic variance explained of each detected QTL was achieved based on the phenotypic variance in the population [65].

## Supplementary Information

**Additional file 1:** **Figures 1**. Haplotype maps for 12 LGs. **Figures 2**. Heat maps for 12 LGs. **Figures 3.** 17 growth-related traits QTLs.

## Data Availability

The data generated or analyzed in this study are included in this article and its supplementary information files. Other materials that support the findings of this study are available from the corresponding author on reasonable request. Not applicable.

## Abbreviations

QTL: Quantitative trait loci
LG: Linage group
SLAF-seq: Specific locus amplified fragment sequencing
LOD: Logarithm of odds
MAS: Marker-assisted selection
SNP: Single nucleotide polymorphism
NGS: Next-generation sequencing
CTAB: Cetyl trimethyl ammonium bromide
CP: Cross-pollination
CV: Coefficients of variation
BC: Backcross
RILs: Recombination Inbred Lines
SSR: Simple sequence repeat
ISSR: Inter-simple sequence repeat
AFLP: Amplified fragment length polymorphism
RAPD: Random amplified polymorphic DNA
