# Supplementary material for: QTL mapping for growth-related traits by constructing the first genetic linkage map in Simao pine
Source: BMC Plant Biol. 2022 Jan 22;22:48. doi: 10.1186/s12870-022-03425-y (PMC8783431; doi:10.1186/s12870-022-03425-y)

Supplementary Material

## Supplementary Figures

Supplementary Figures 1: Haplotype maps for 12 LGs

1.1 Haplotype map for LG1


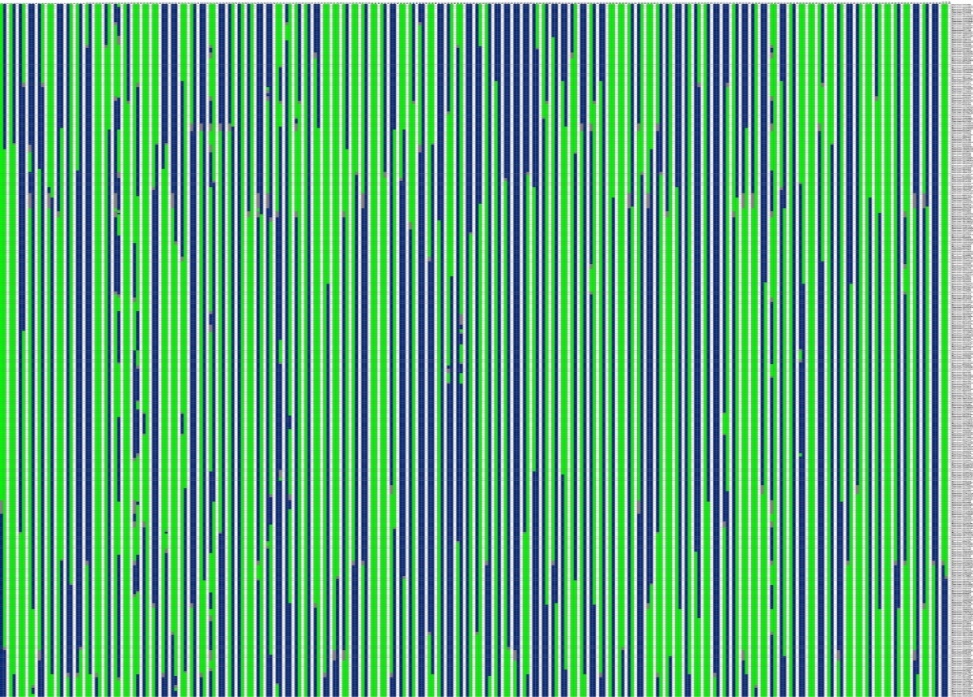


1.2 Haplotype map for LG2


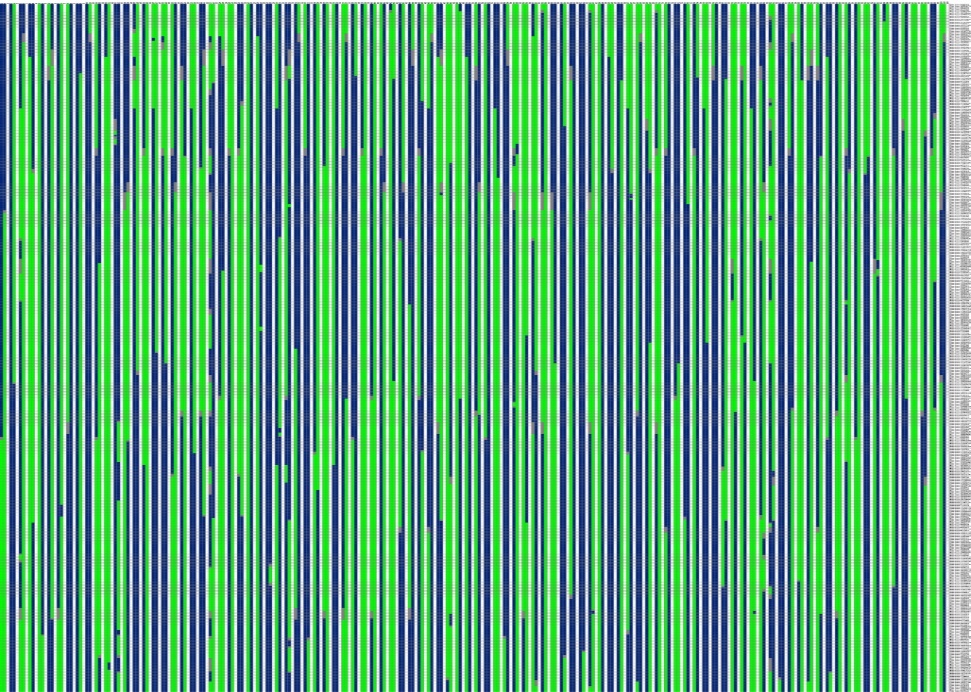


1.3 Haplotype map for LG3


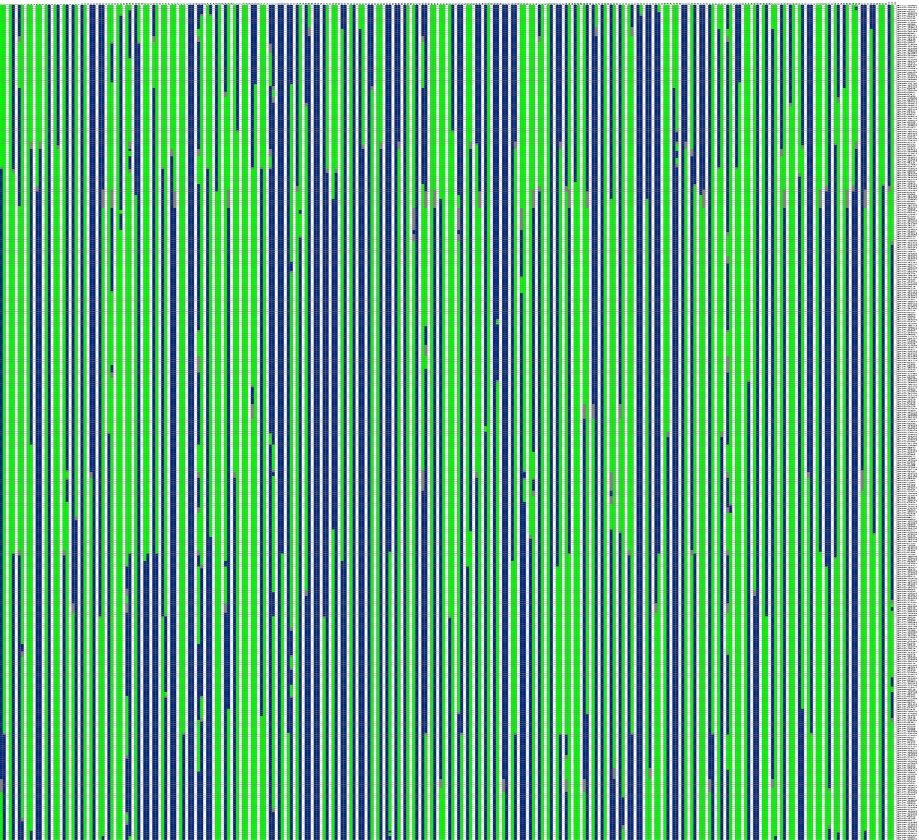


1.4 Haplotype map for LG4


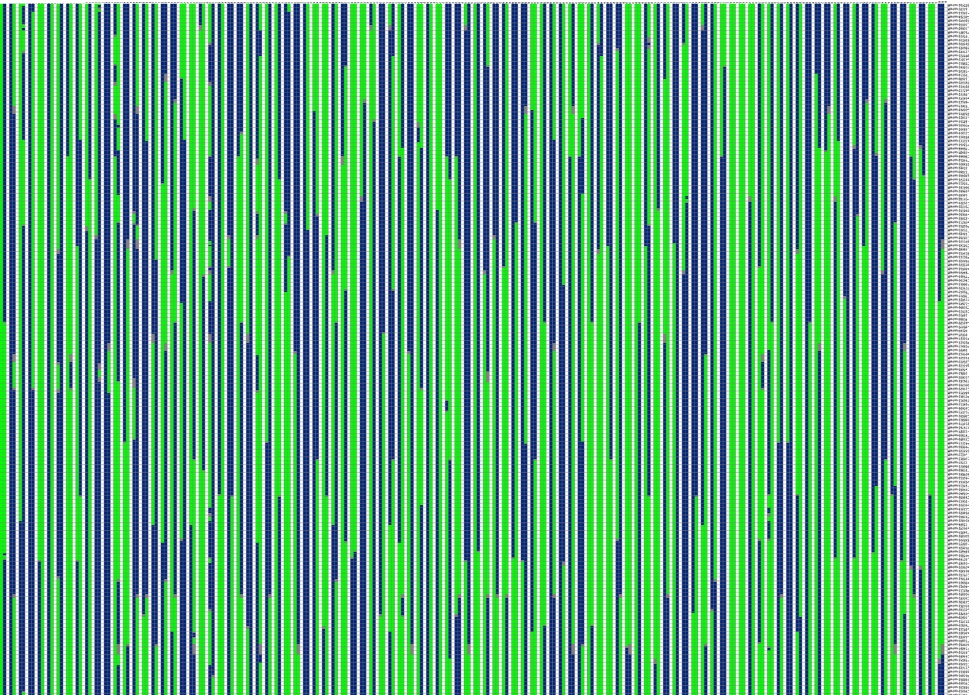


1.5 Haplotype map for LG5


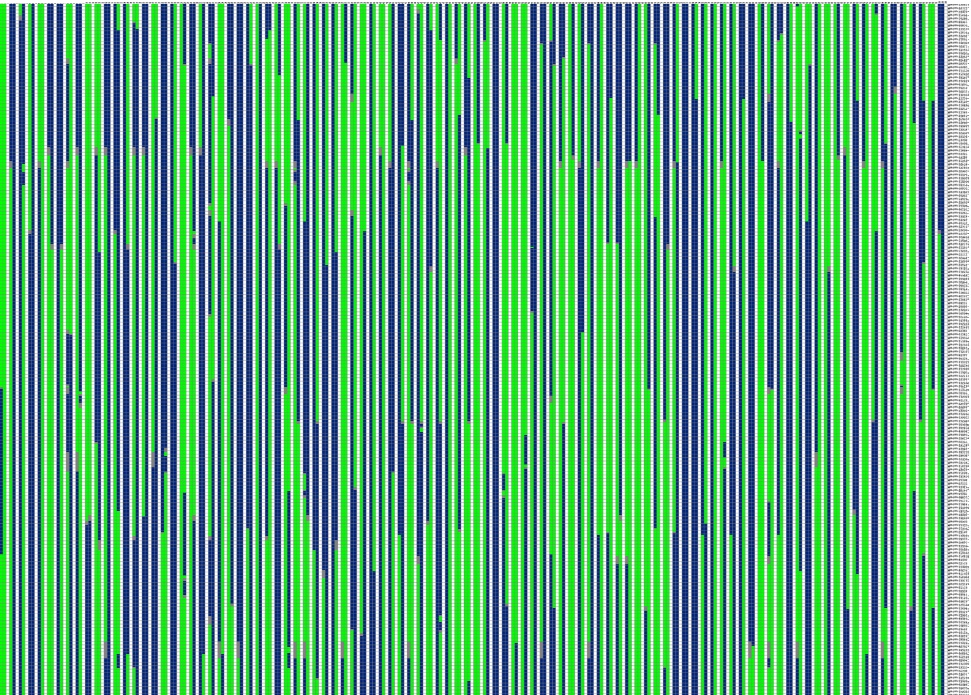


1.6 Haplotype map for LG6


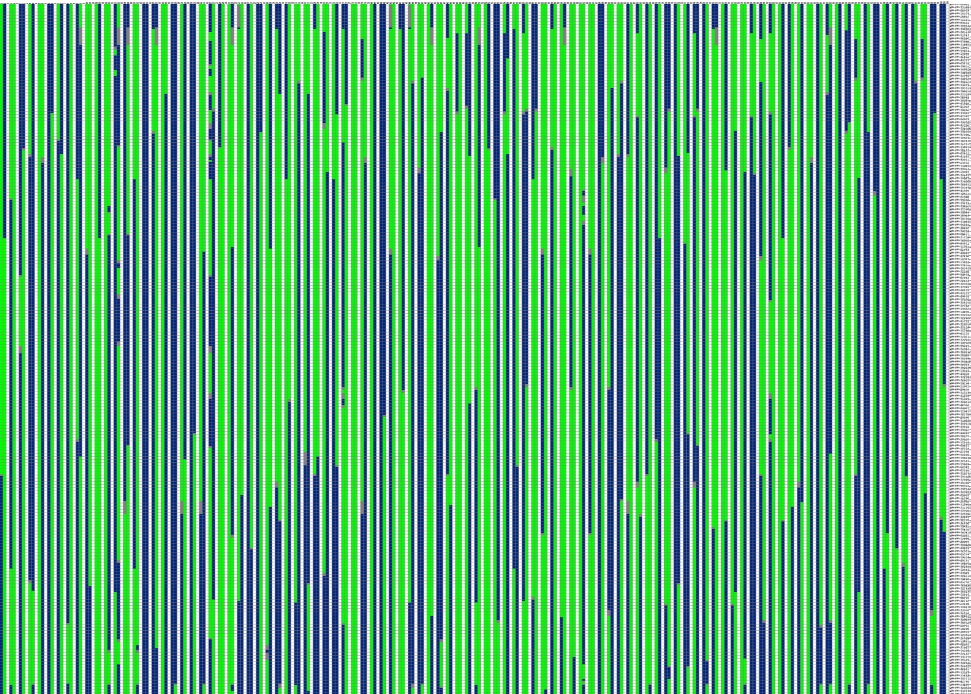


1.7 Haplotype map for LG7


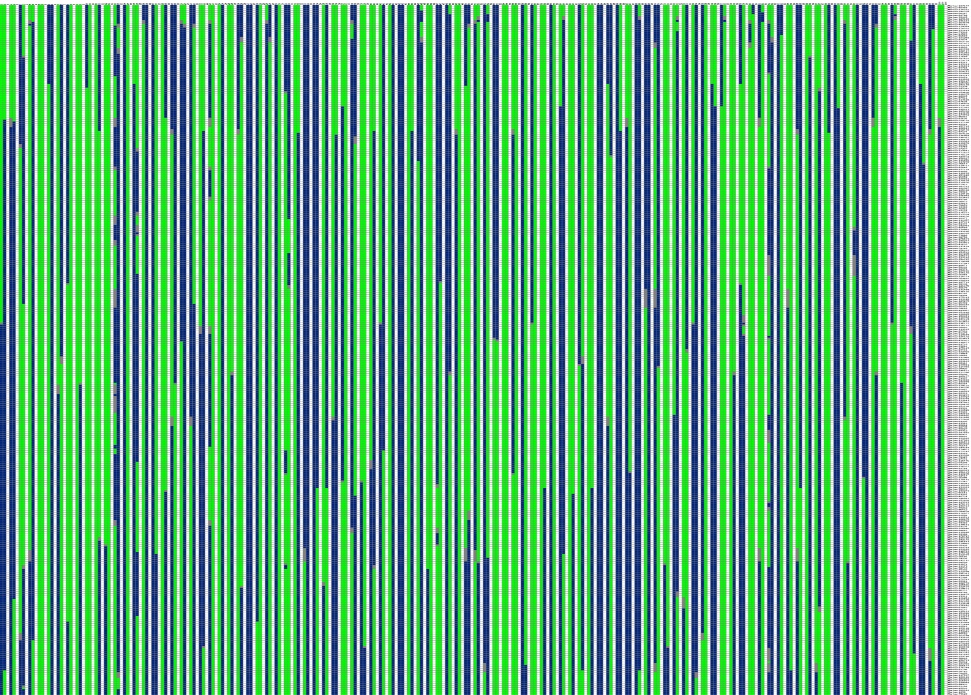


1.8 Haplotype map for LG8


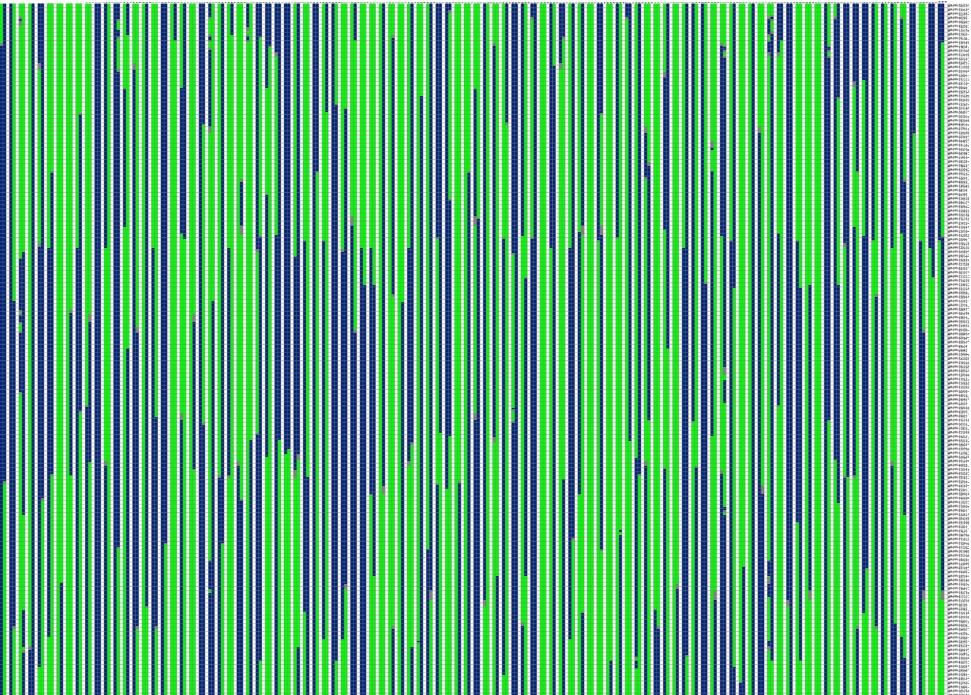


1.9 Haplotype map for LG9


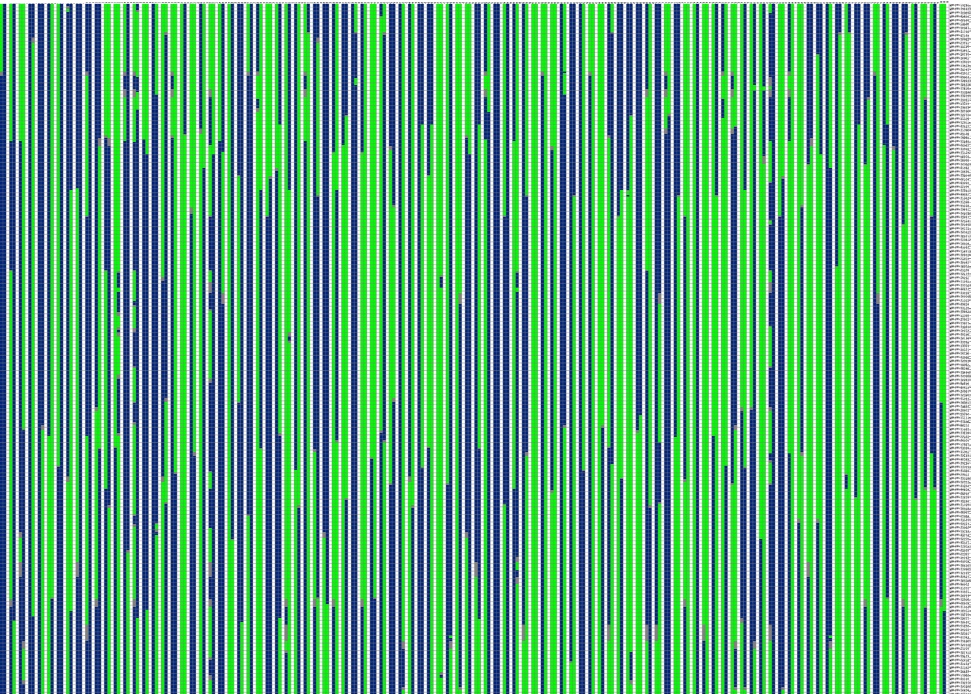


1.10 Haplotype map for LG10


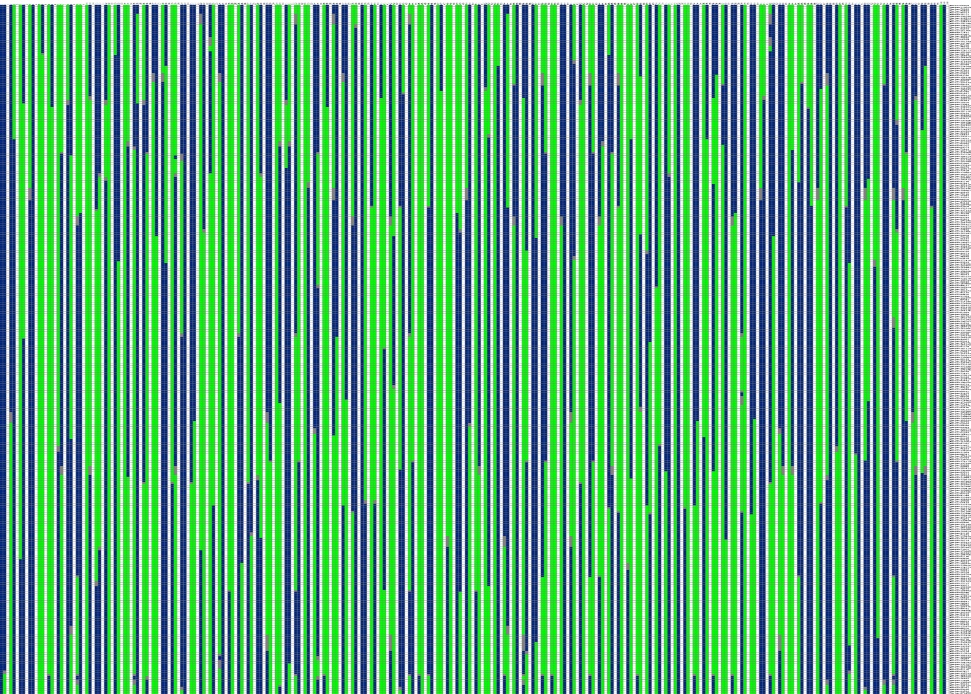


1.11 Haplotype map for LG11


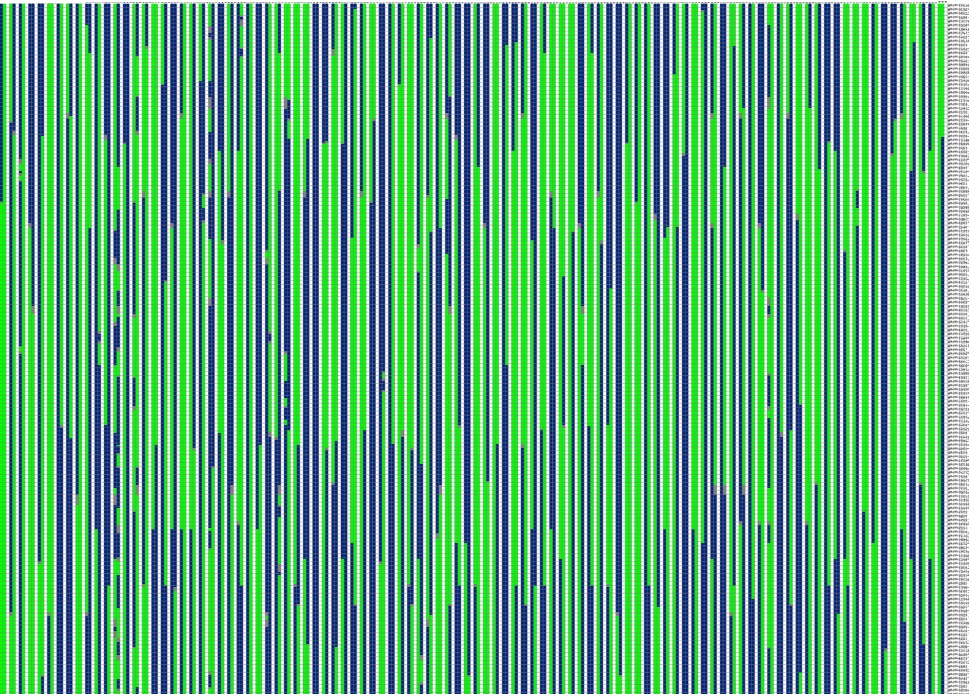


1.12 Haplotype map for LG12


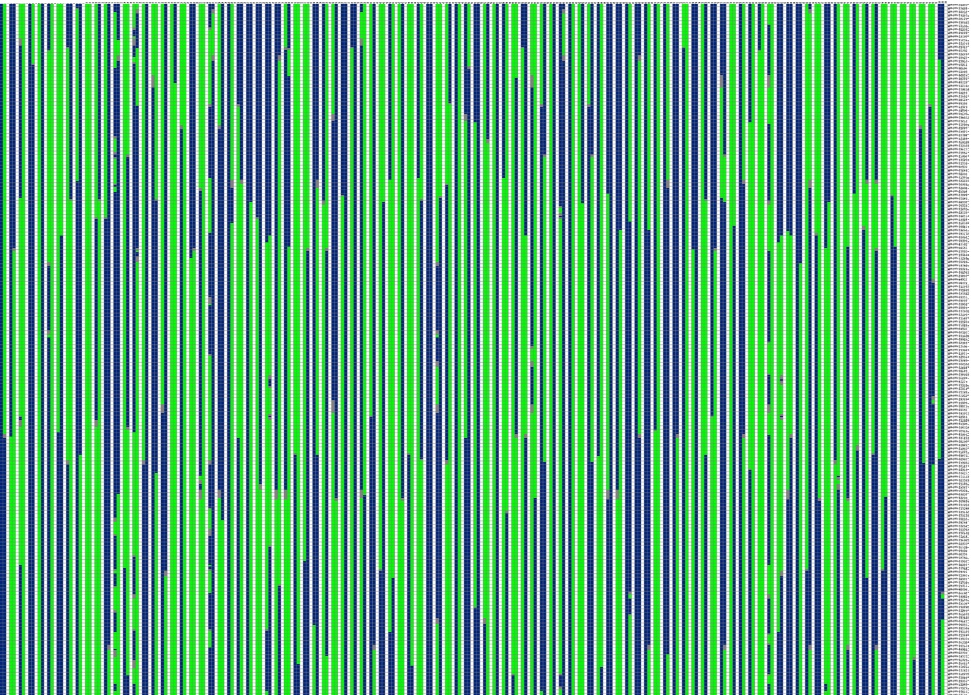


Supplementary Figures 2: Heat maps for 12 LGs

2.1 Heat map for LG1


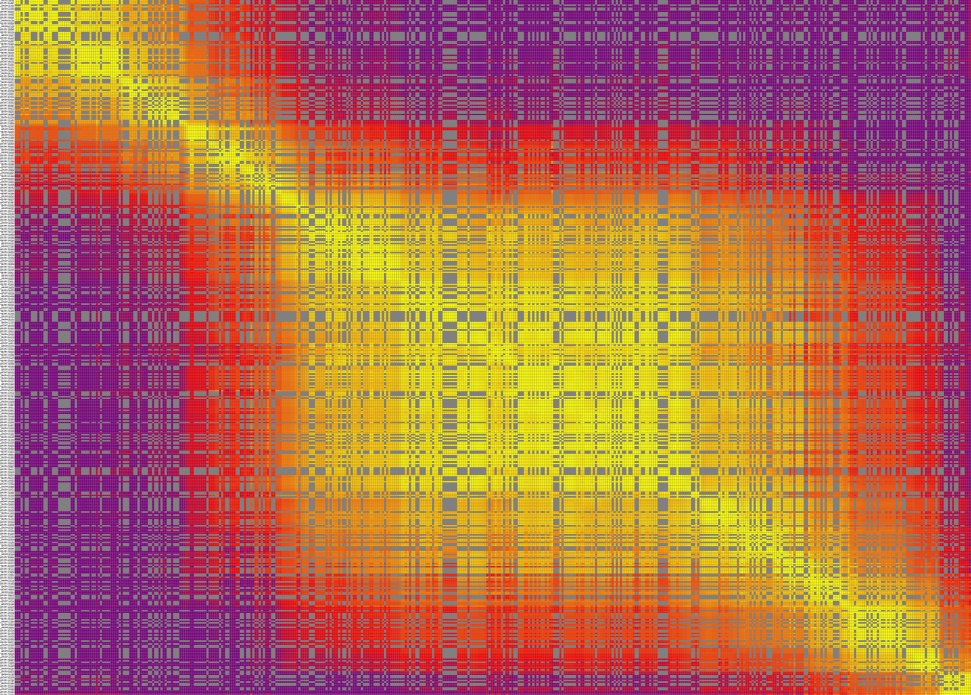


2.2 Heat map for LG2


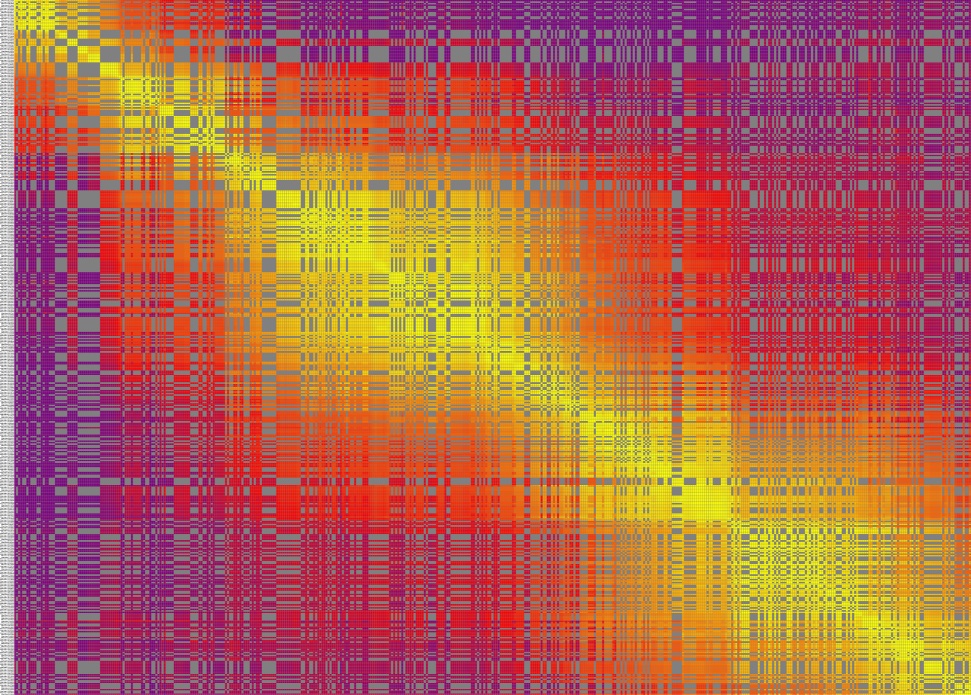


2.3 Heat map for LG3


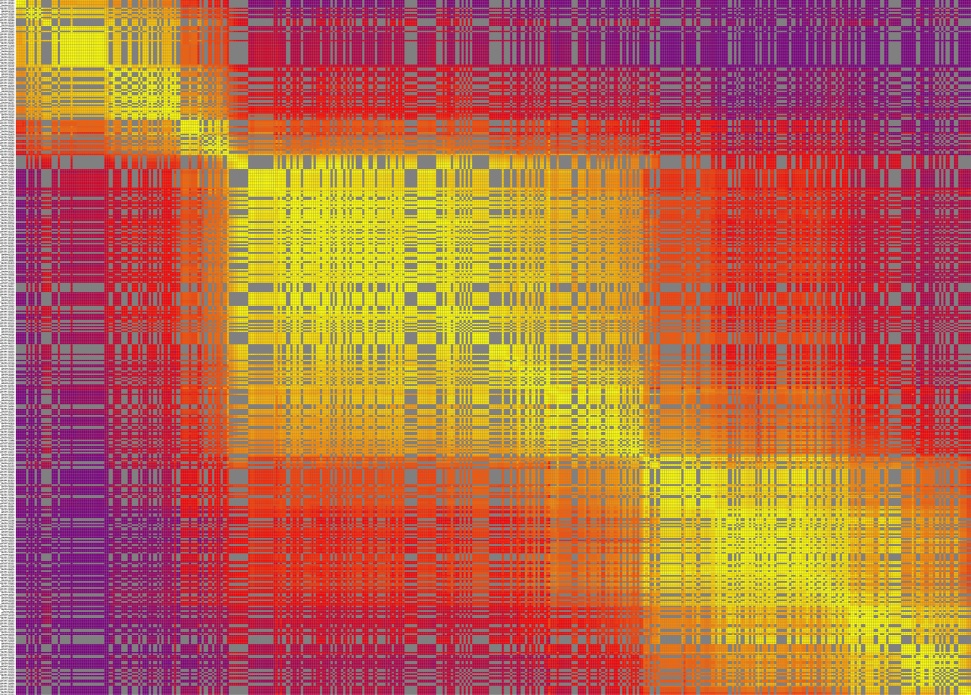


2.4 Heat map for LG4


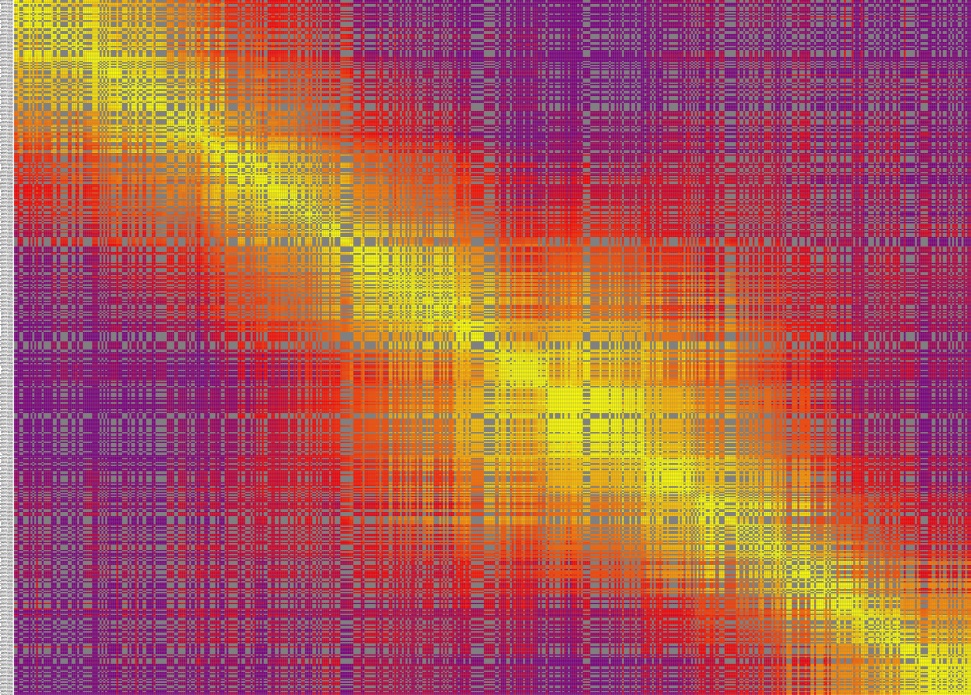


2.5 Heat map for LG5


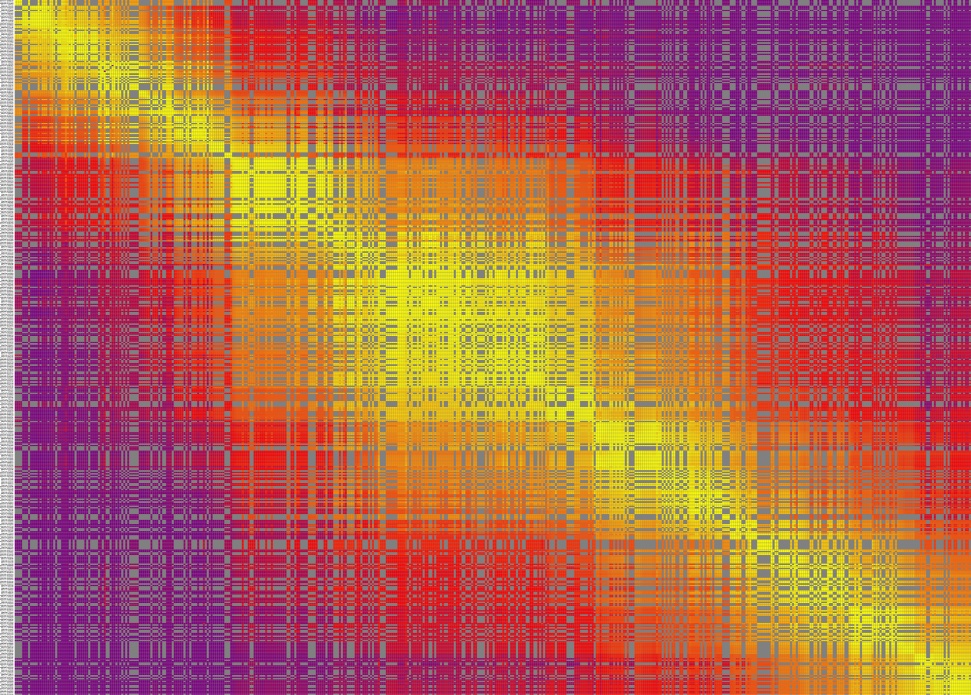


2.6 Heat map for LG6


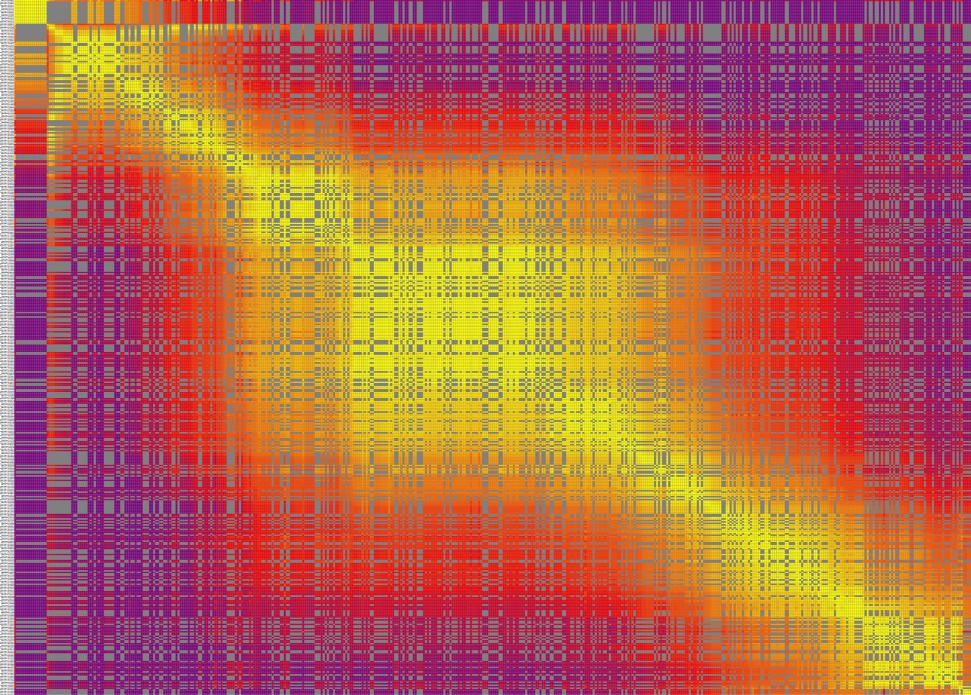


2.7 Heat map for LG7


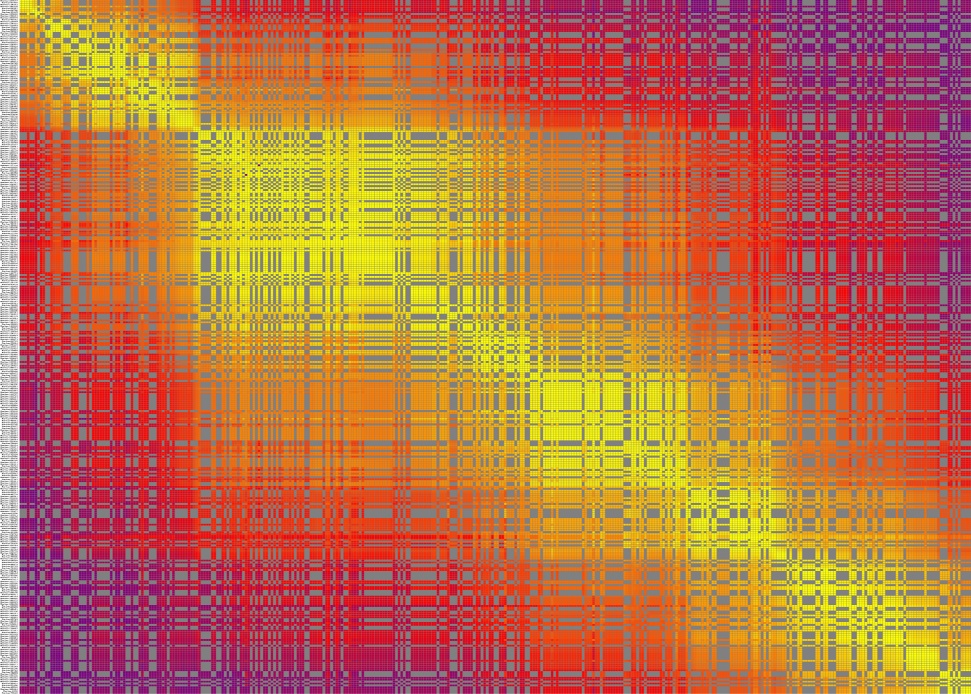


2.8 Heat map for LG8


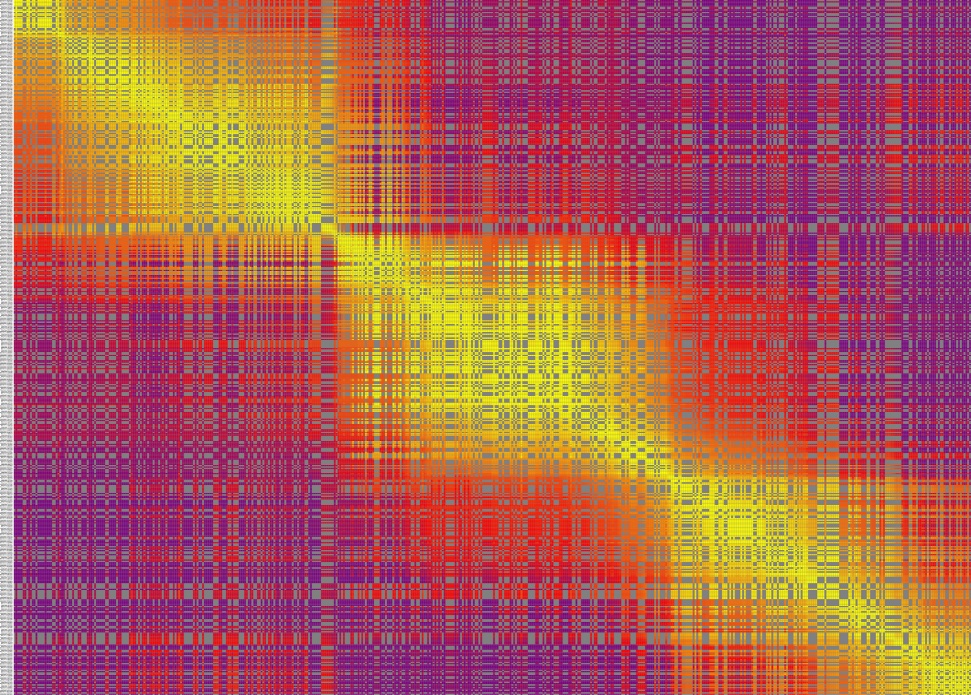


2.9 Heat map for LG9


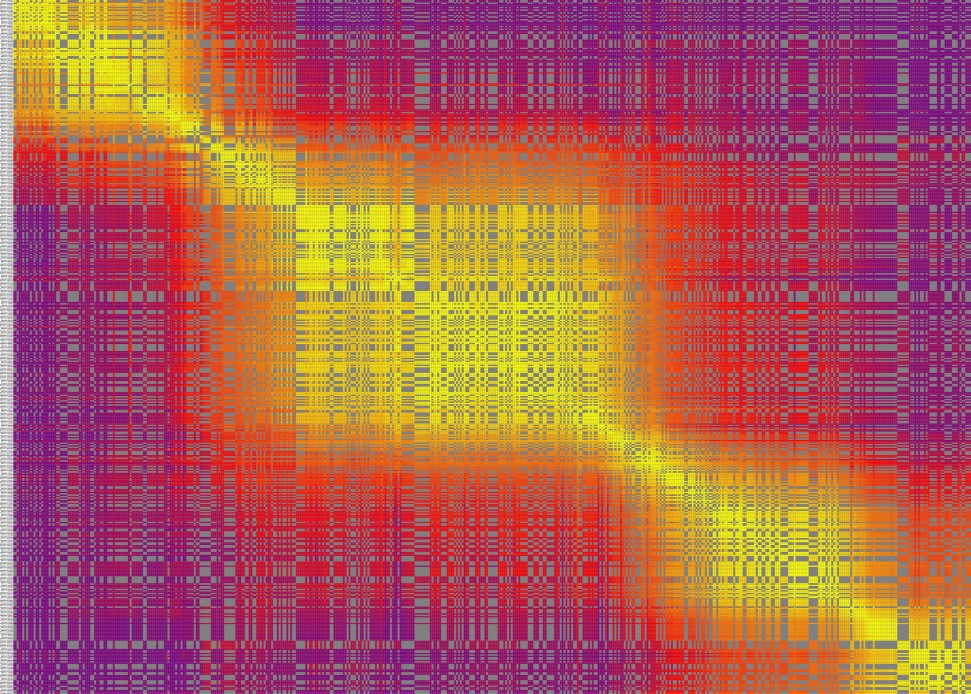


2.10 Heat map for LG10


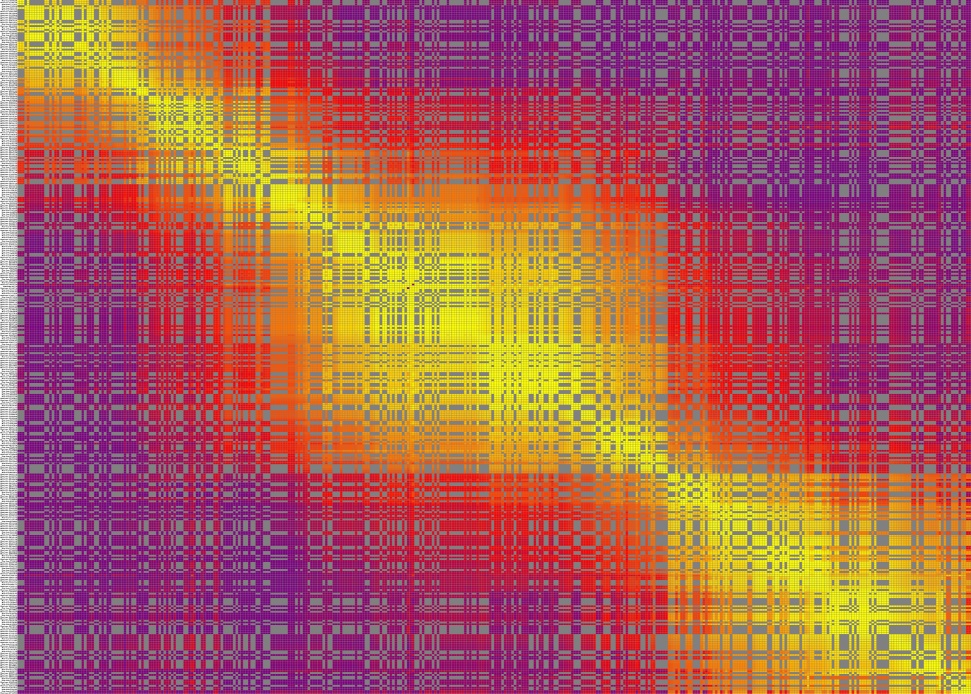


2.11 Heat map for LG11


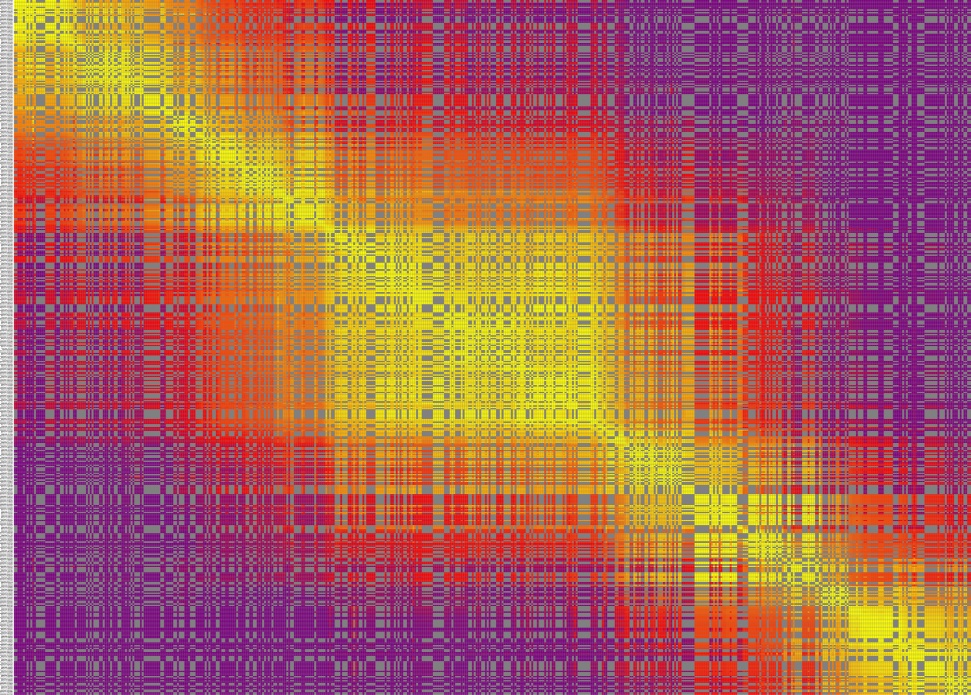


2.12 Heat map for LG12


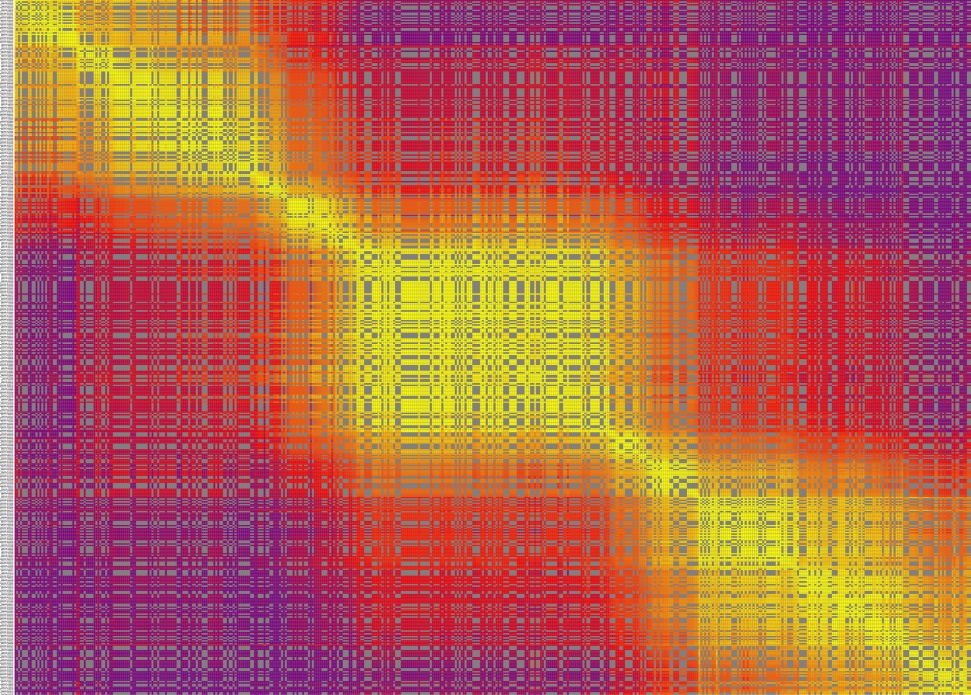


Supplementary Figures 3: 17 growth-related traits QTLs

3.1 Dh16.1 in LG3


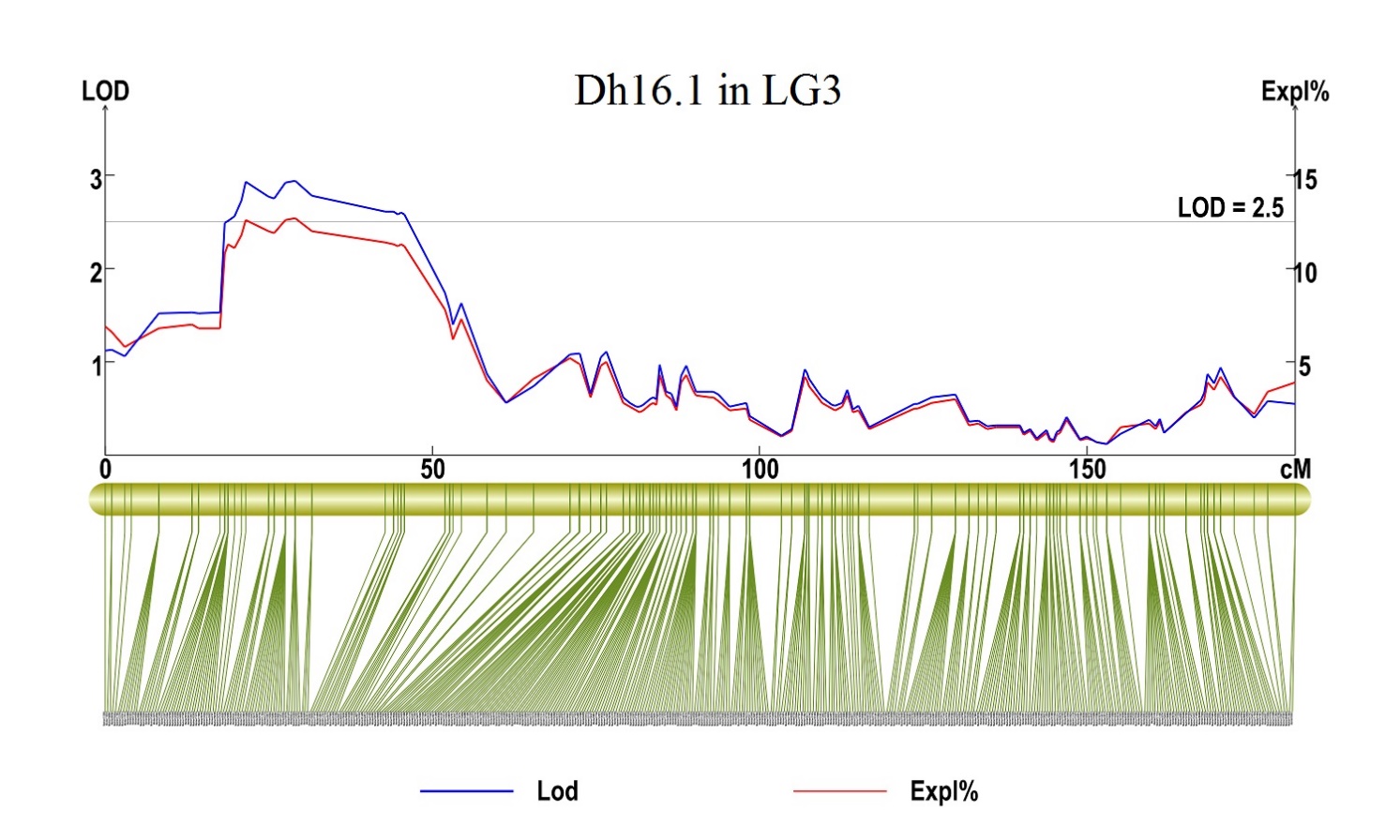


3. 2 Dh16.2 in LG10


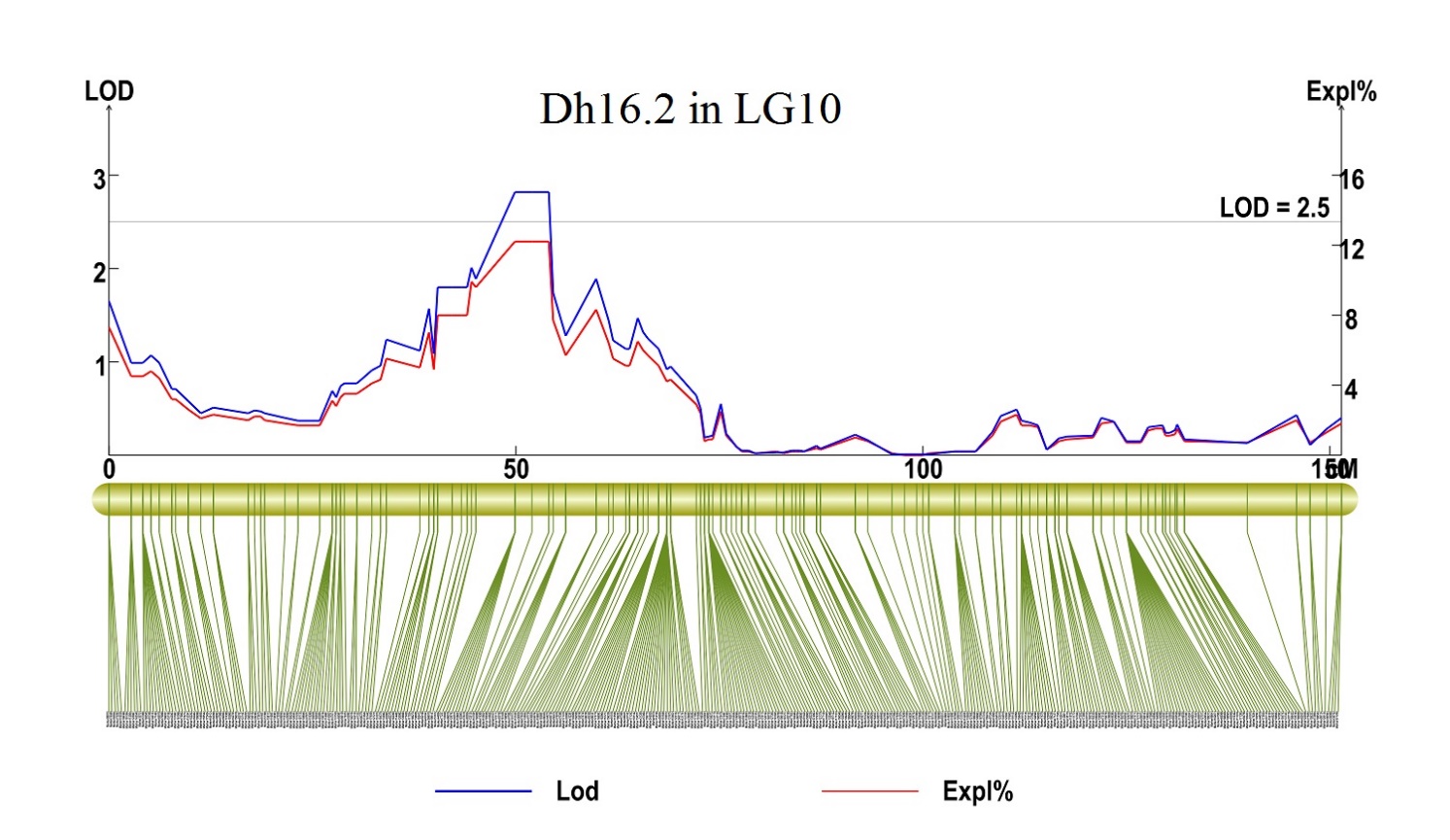


3. 3 Dh17.1 in LG9


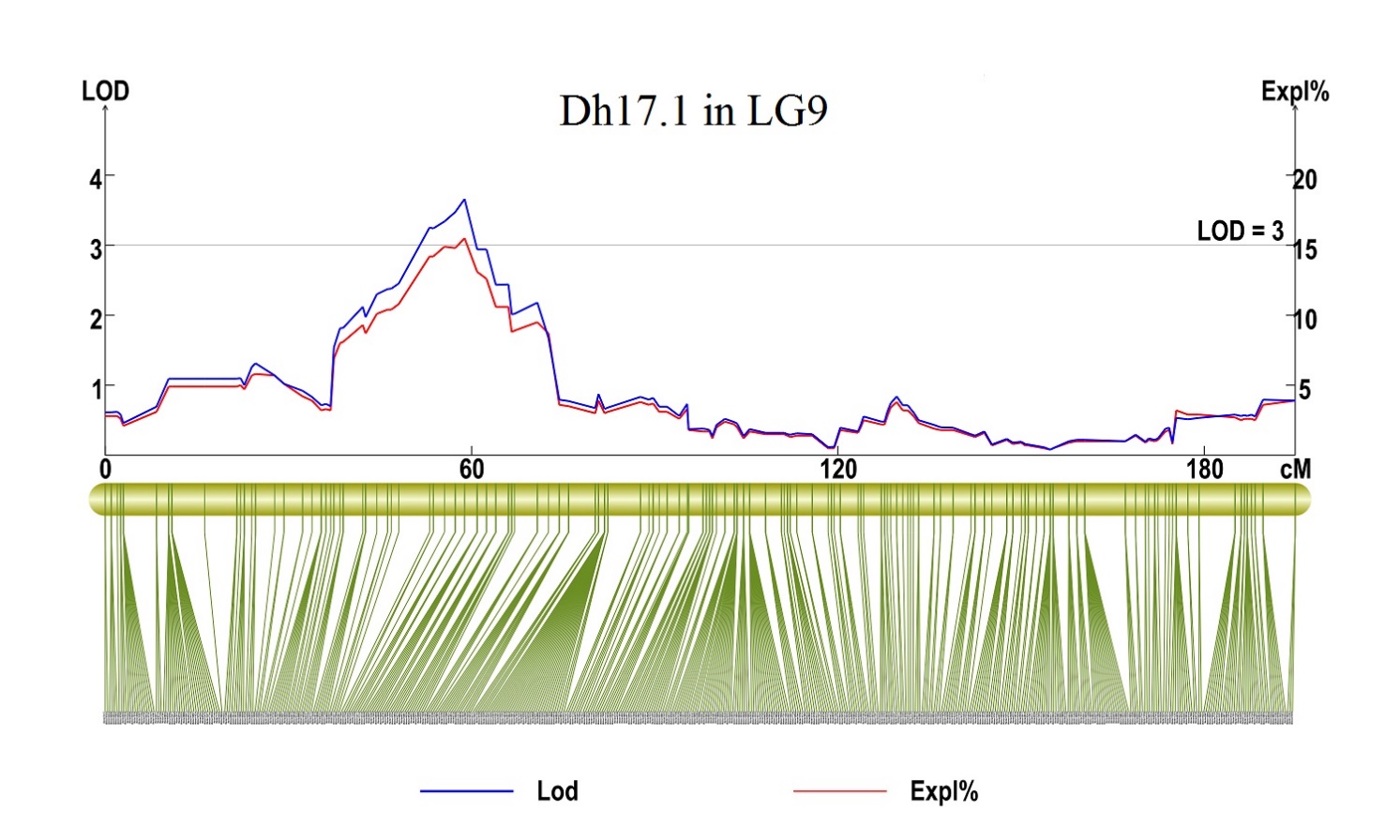


3.4 Dh18.1 and Dh 18.2 in LG11


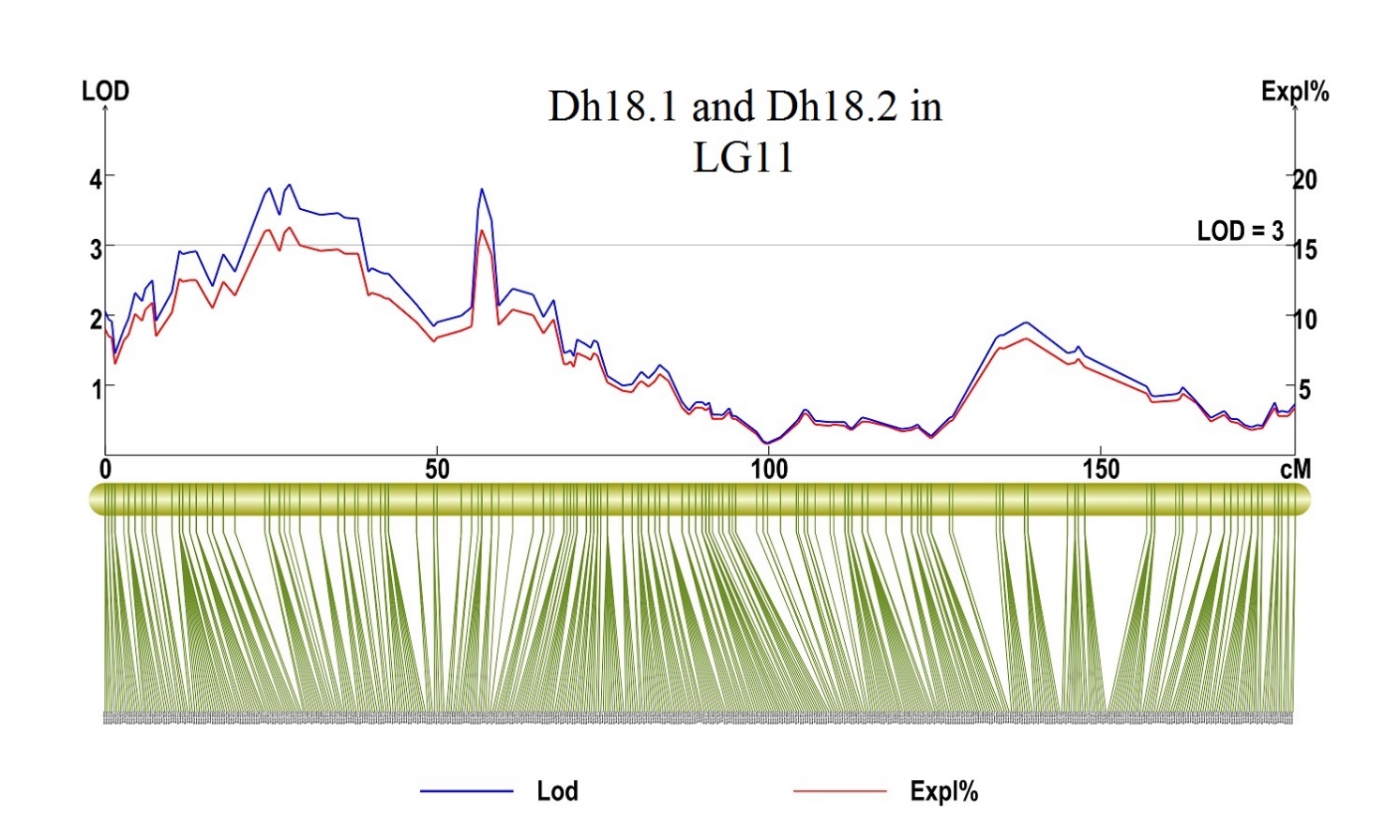


3.5 Dh18.3 in LG12


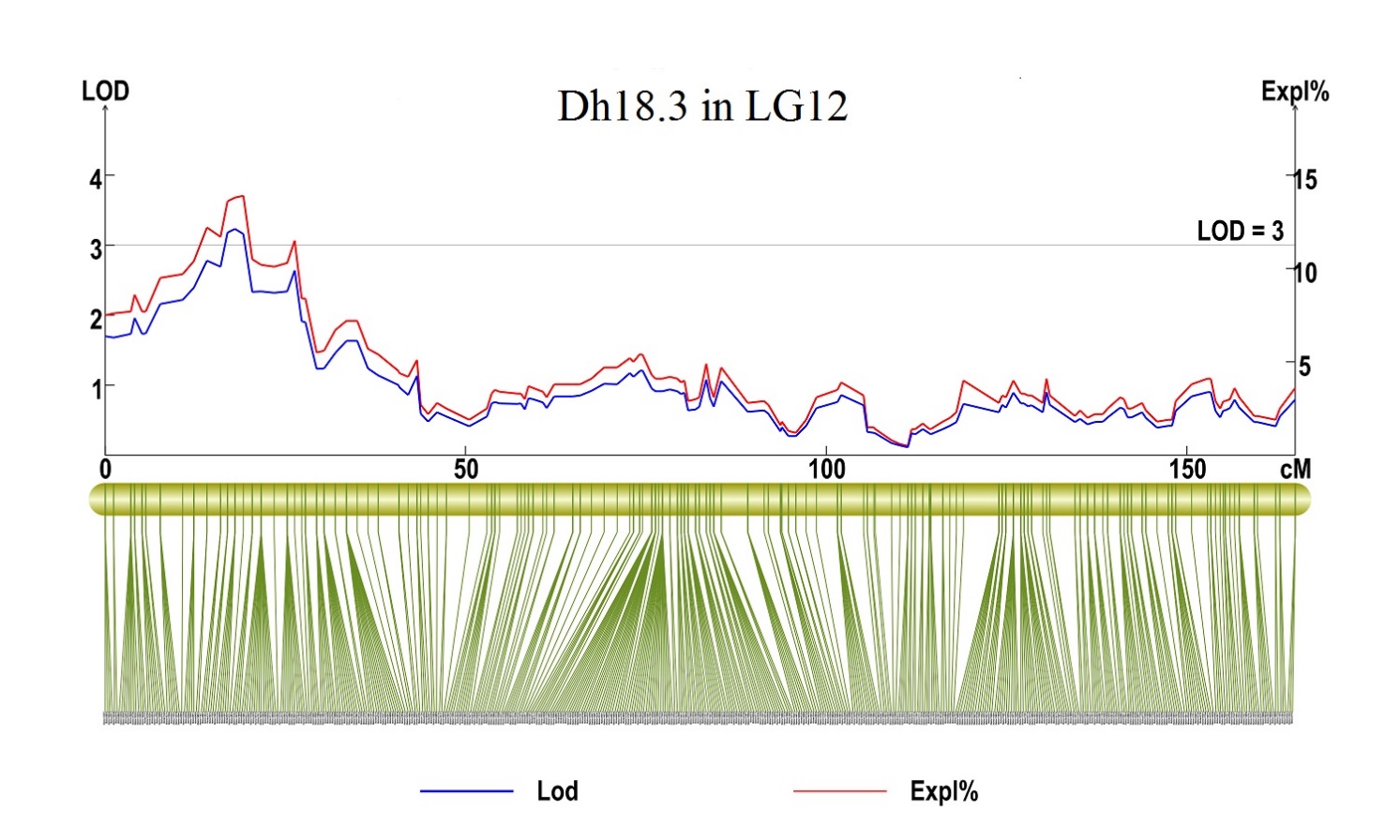


3.6 Dbd17.1 in LG3


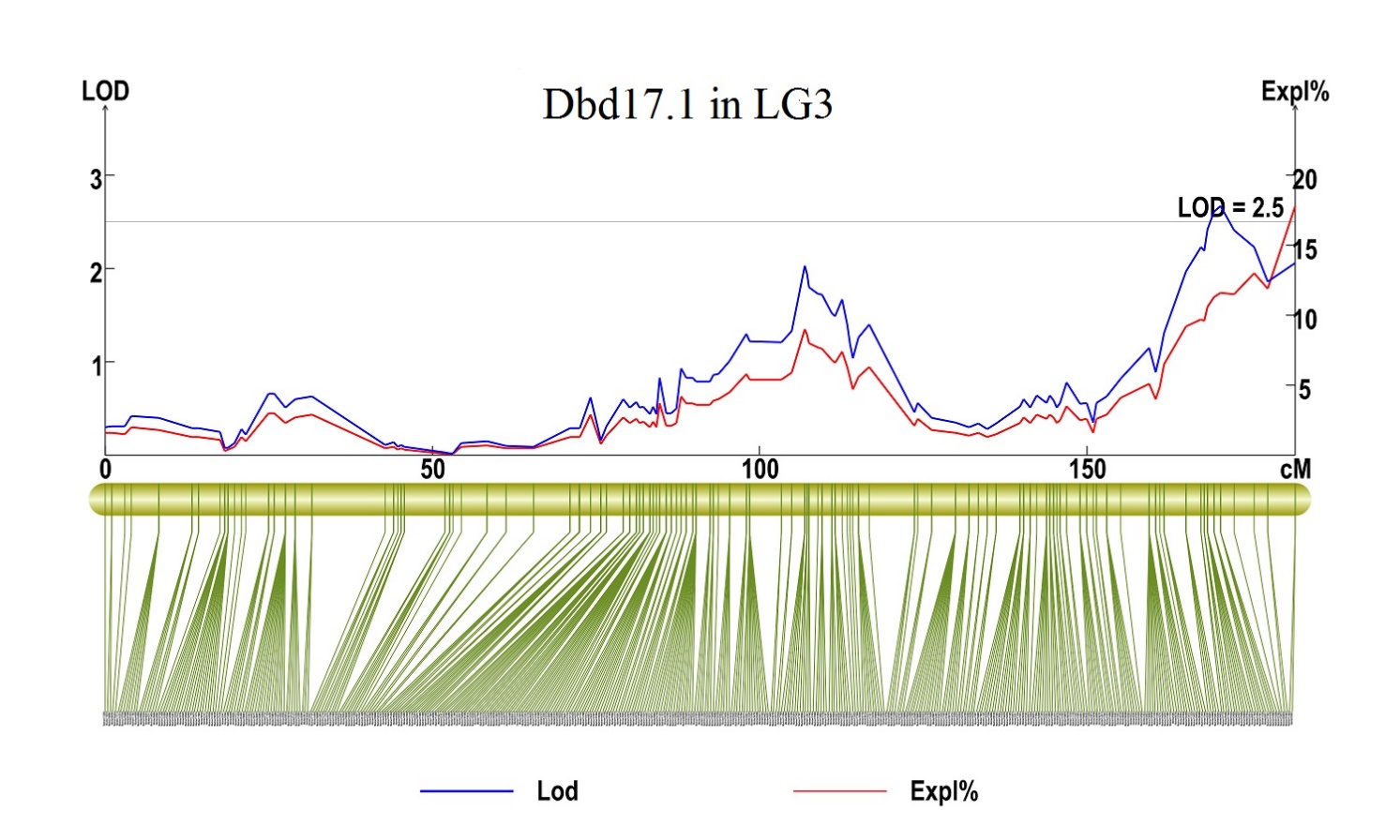


- 1. Dbd17.2 in LG4


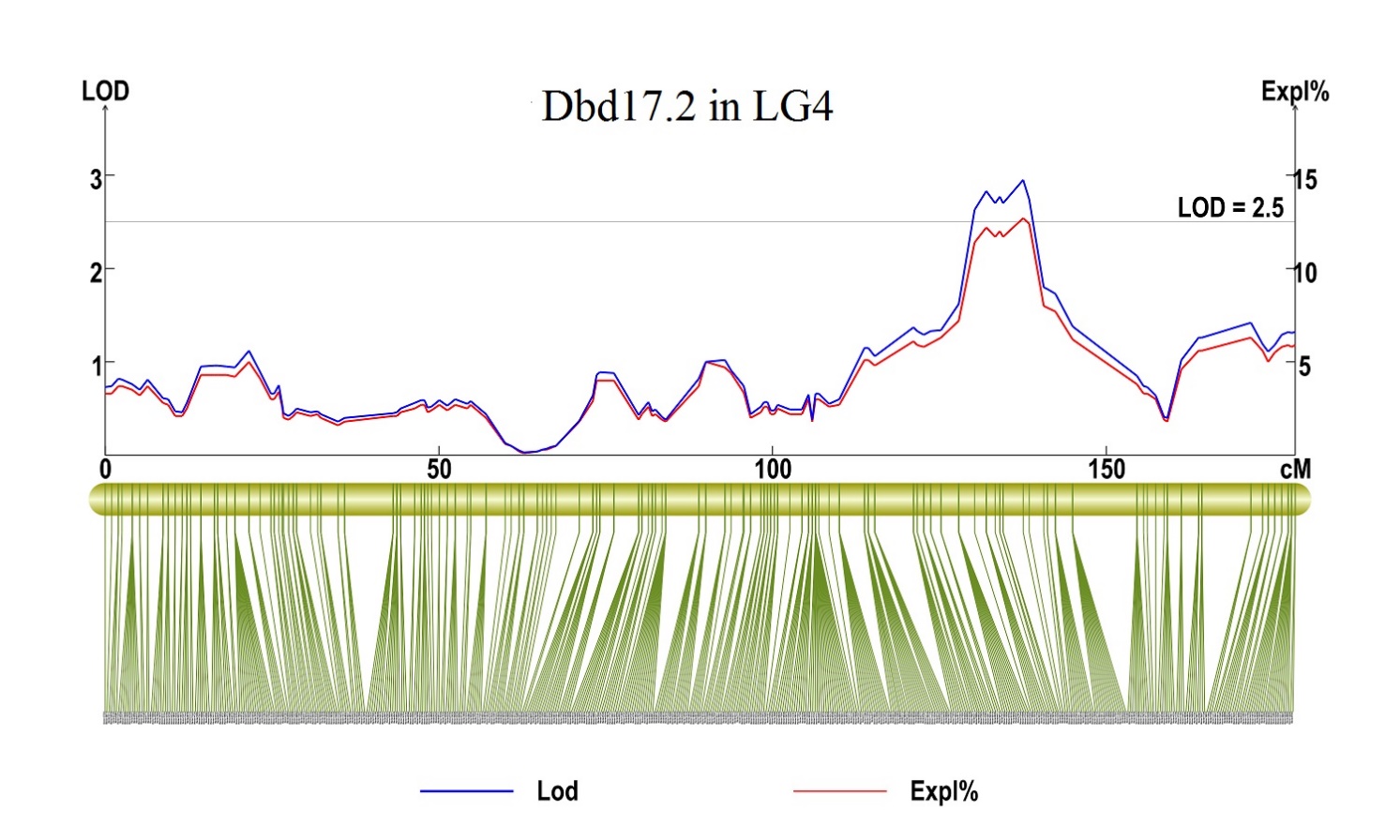


- 1. Dbd17.3, Dbd17.4 and Dbd17.5 in LG6


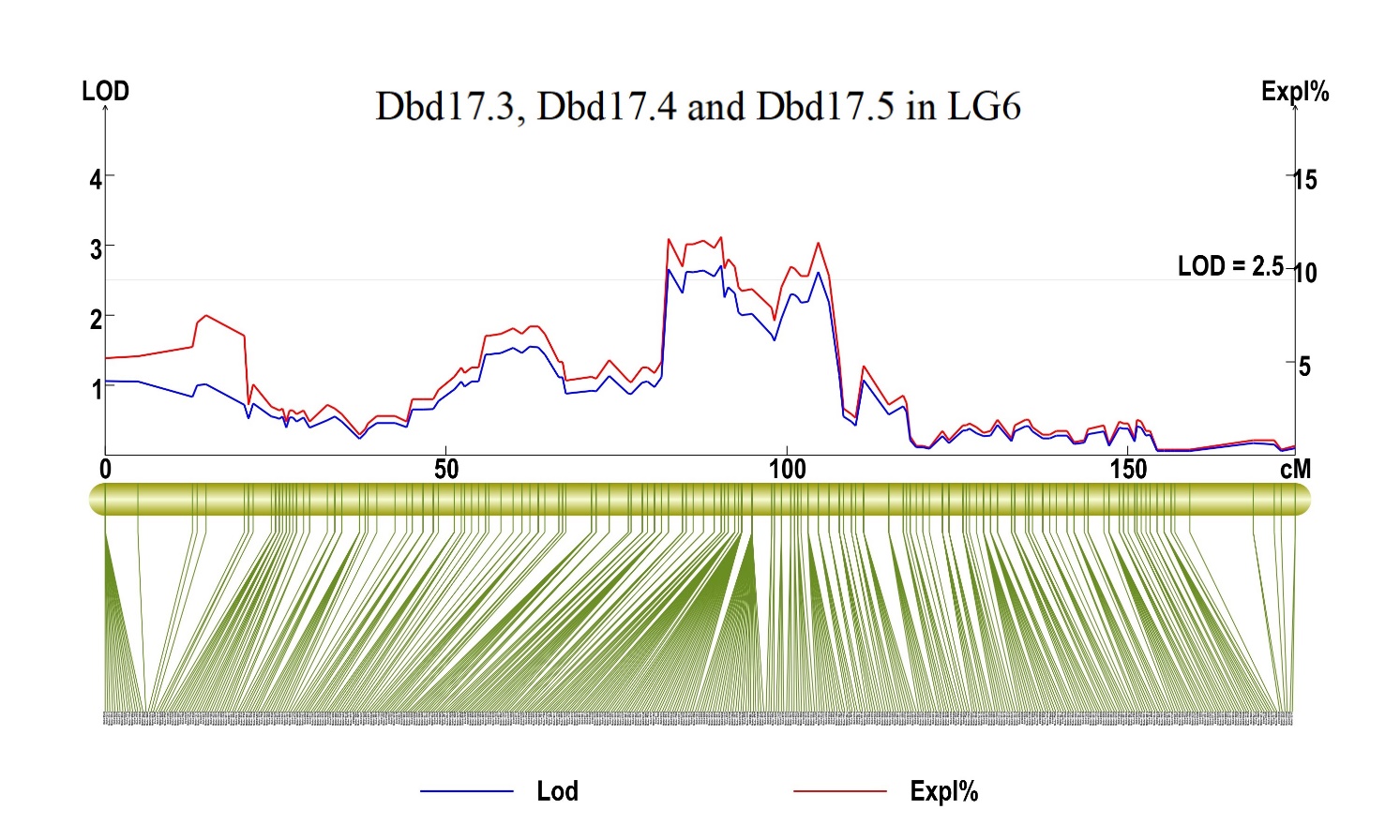


3. 9 Dn17.3, Dnl17.2 and Dnl17.3 in LG1


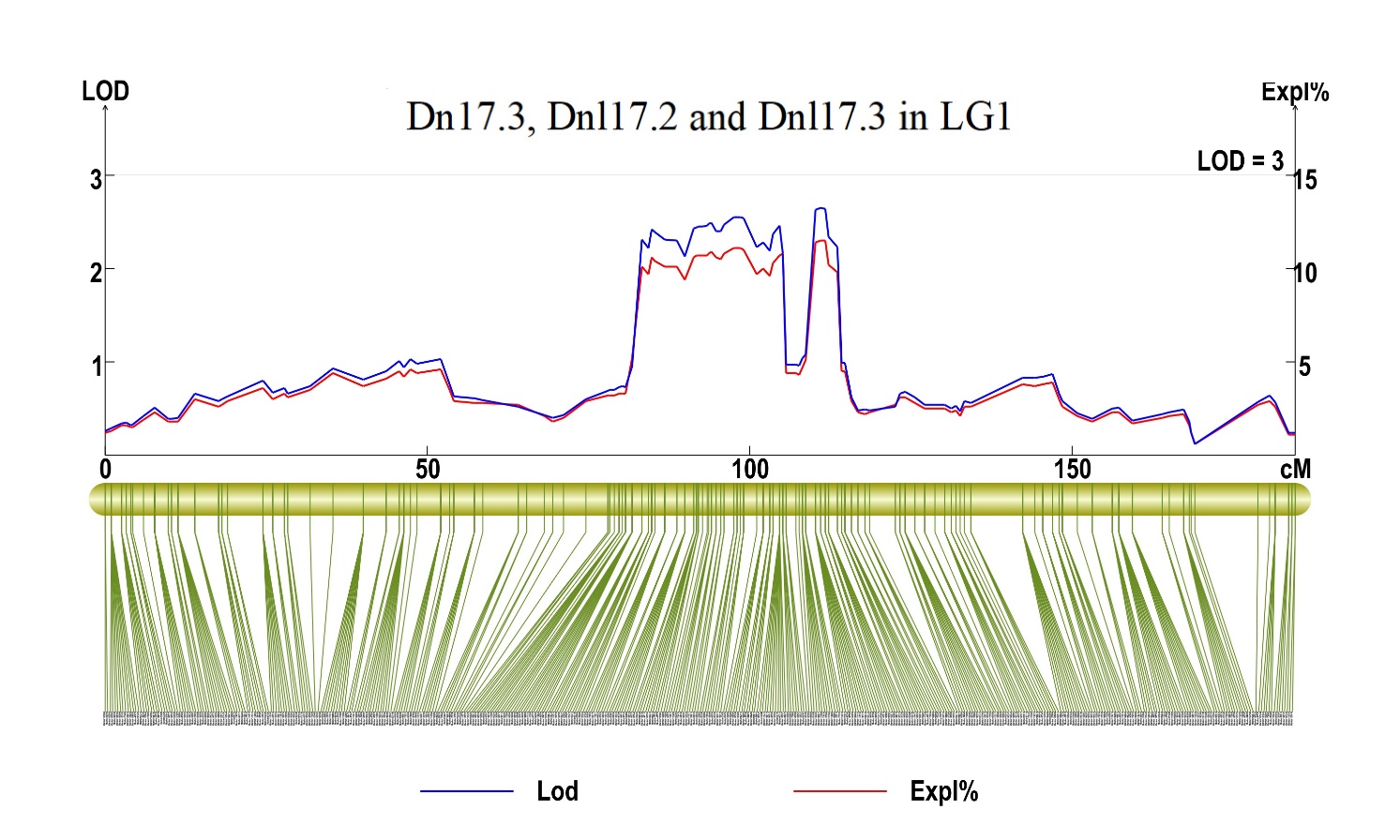


3. 10 Dnl18.1 in LG12


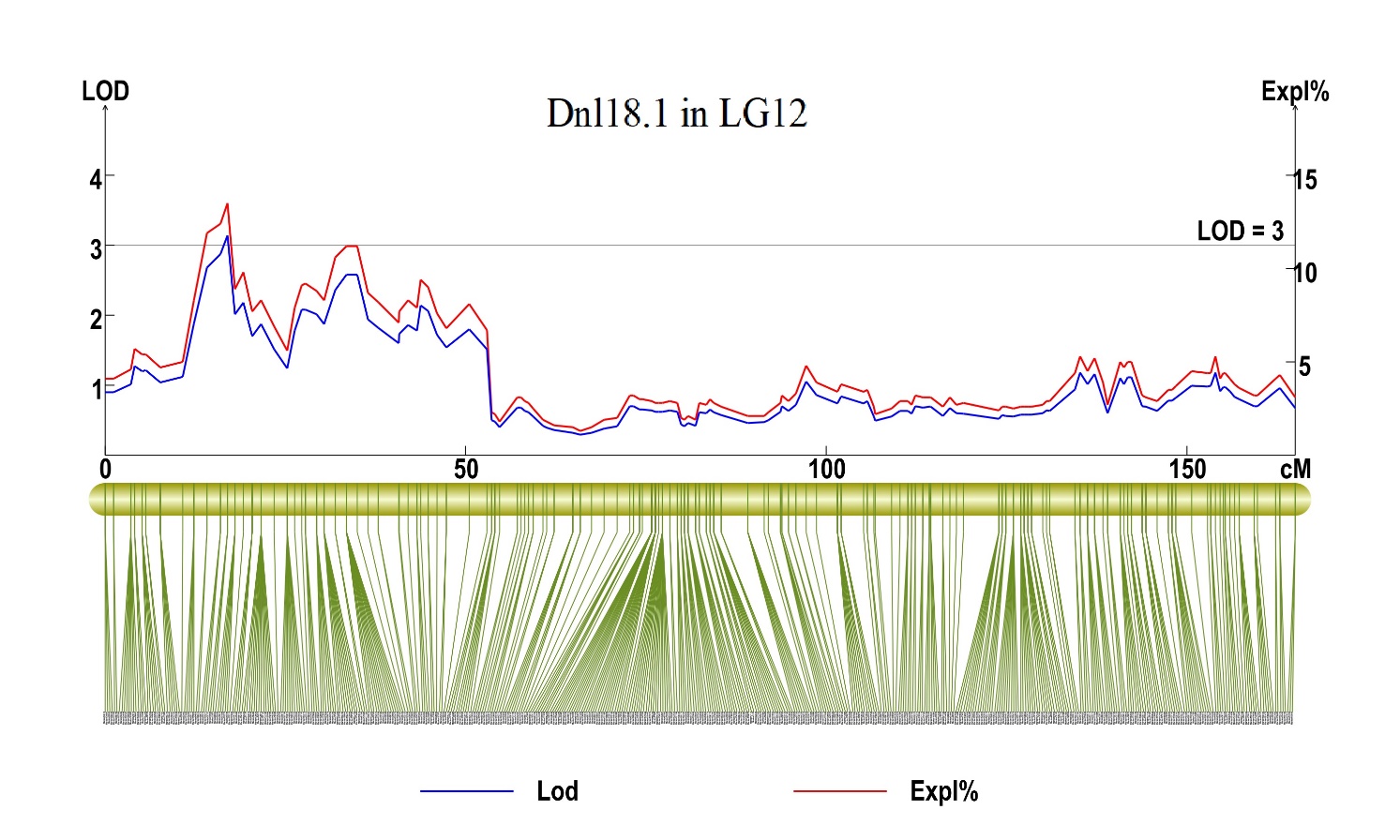


3. 11 Dnd17.1 in LG4


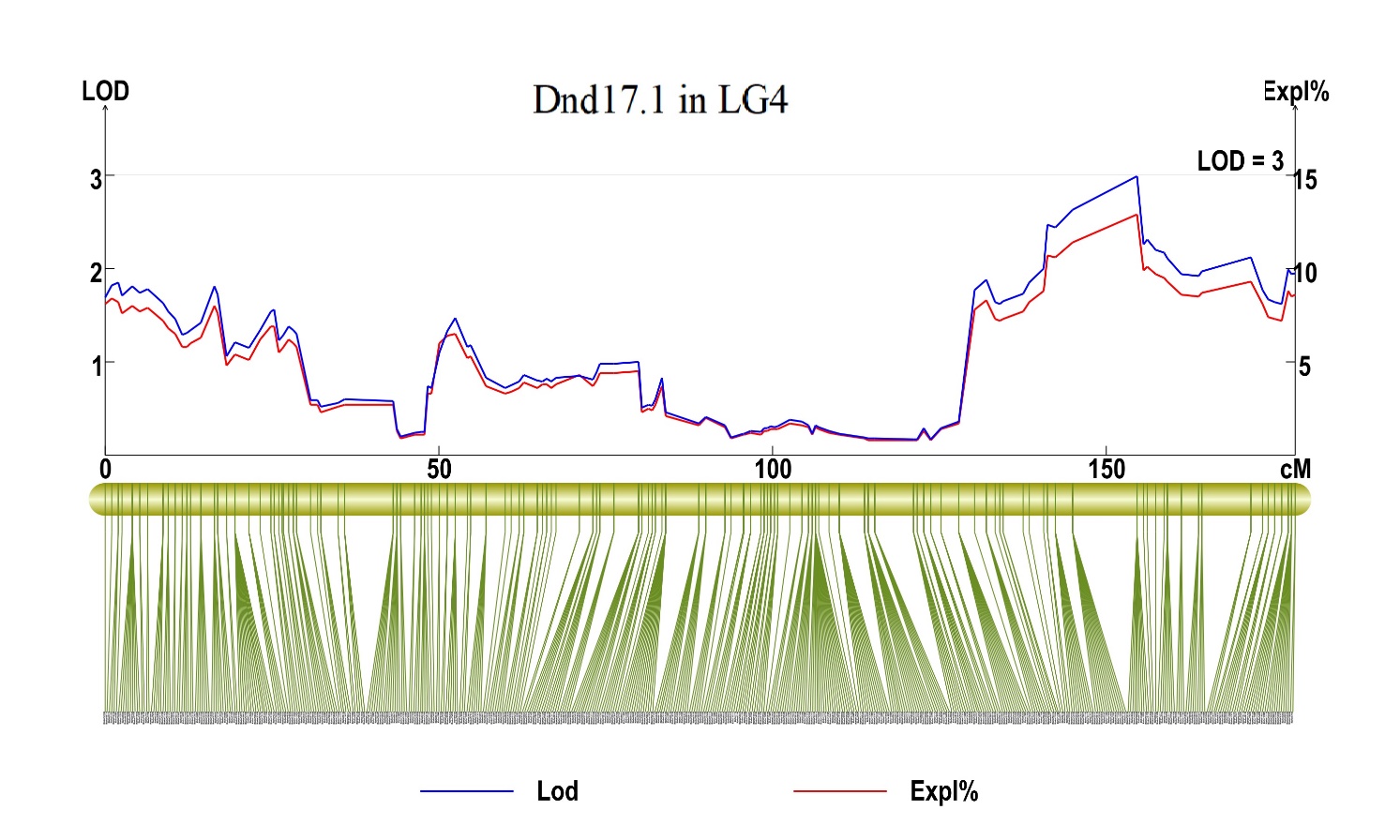


3. 12 Dnd18.1 in LG4


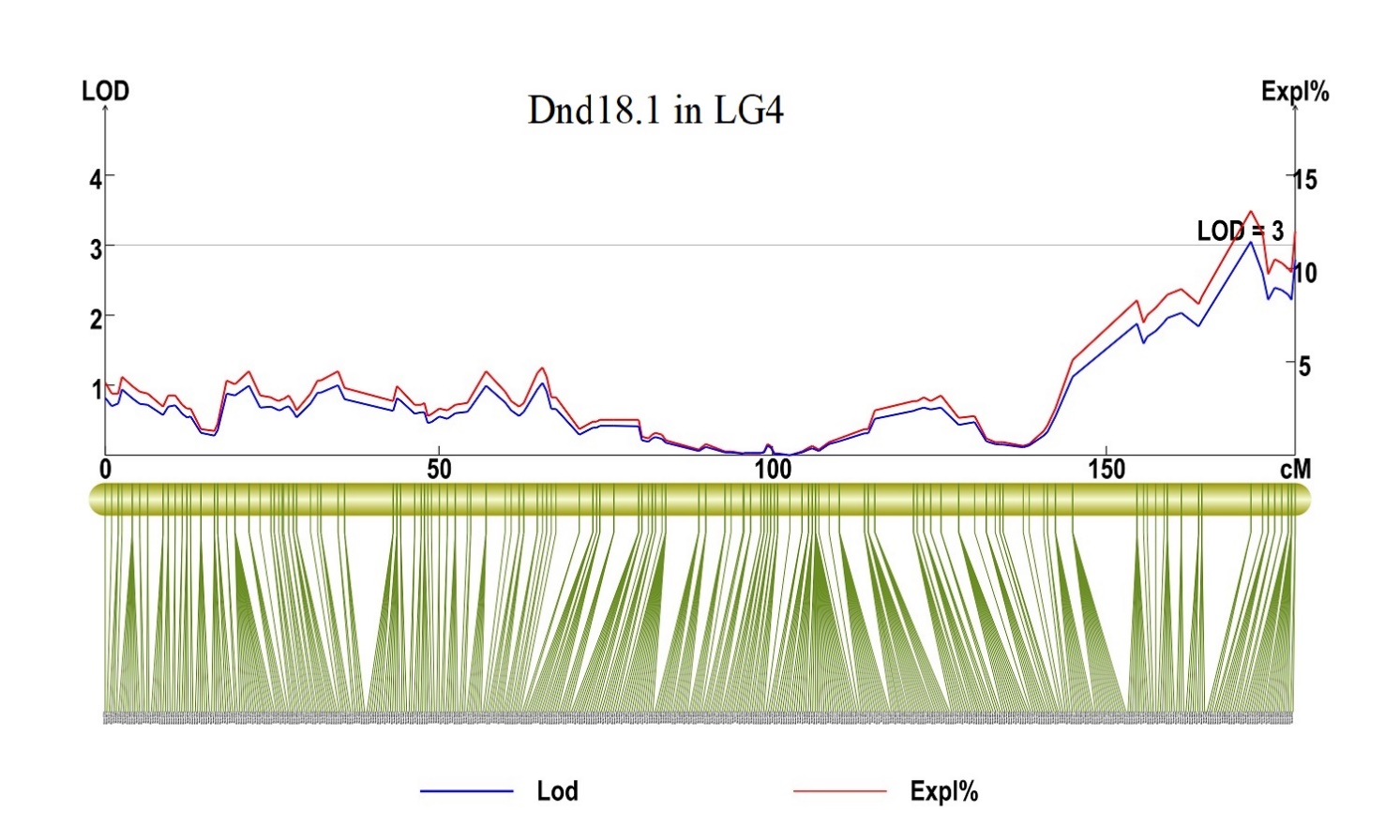

Supplement: Supplementary file 1 — Additional file 1: Figures 1. Haplotype maps for 12 LGs. Figures 2. Heat maps for 12 LGs. Figures 3. 17 growth-related traits QTLs. [file 12870_2022_3425_MOESM1_ESM.docx]
